# Synthesis and self-assembly of 1-deoxyglucose derivatives as low molecular weight organogelators

**DOI:** 10.3762/bjoc.7.31

**Published:** 2011-02-21

**Authors:** Guijun Wang, Hao Yang, Sherwin Cheuk, Sherman Coleman

**Affiliations:** 1 Department of Chemistry, University of New Orleans, New Orleans, LA 70148, Phone: 504 280-1258, Fax: 504 280-6860; 2 Dillard University, 2601 Gentilly Boulevard, New Orleans, Louisiana 70122

**Keywords:** 1,5-anhydroglucitol, carbohydrate, hydrogelator, organogelator, self-assembly

## Abstract

Low molecular weight gelators are an important class of molecules. The supramolecular gels formed by carbohydrate derived low molecular weight gelators are interesting soft materials that show great potential for many applications. Previously, we have synthesized a series of methyl 4,6-*O*-benzylidene-α-D-glucopyranoside derivatives and found that several of them are good gelators for water, aqueous mixtures of DMSO, or aqueous mixtures of ethanol. The gelation efficiency of these glycolipid derivatives is dependent upon the structures of their acyl chains. In order to understand the influence of the anomeric position of the sugar headgroup towards self-assembly, we synthesized a series of 1-deoxyglucose analogs, and examined their gelation properties in several solvents. Several long chain esters, including diacetylene containing esters, and aryl esters exhibited gelation in ethanol, aqueous ethanol, or aqueous DMSO. The synthesis and characterization of these novel analogs are reported.

## Introduction

In recent years, the field of low molecular weight gelators (LMWGs) has received a great deal of attention. LMWGs are an interesting class of small molecules that can form reversible supramolecular gels in organic solvents or aqueous solutions [1–9]. Non-covalent interactions such as hydrogen bonding, hydrophobic interactions, and π–π stacking are the main driving forces for the self-assembly of the gelators into 3-dimensional networks. The resulting gels may find applications as soft materials for drug delivery, enzyme immobilization, scaffolds for tissue engineering, etc. [10–14]. The structures of LMWGs span a diverse range; carbohydrates have frequently been used in the synthesis of LMWGs because they are naturally abundant and possess multiple chiral centers that can be selectively functionalized [15–37]. Sugar-based supramolecular hydrogels are being explored as biocompatible soft materials and as matrices for enzymes, DNA, and drug delivery systems [22–28]. Glucose, in particular, is a versatile building block for the preparation of various small molecule gelators [30–37], as it is relatively easy to obtain substituted products by selective functionalization of the anomeric position and the 4- and 6-hydroxy groups. We have found that further derivatization of the glucose headgroup to form different glycolipids can result in organogelators [34–37].

**Figure 1:**
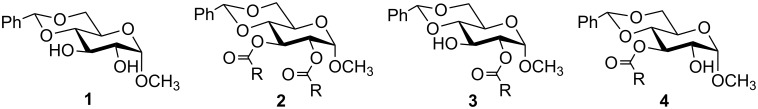
Structures of three ester derivatives of compound **1**.

**Figure 2:**
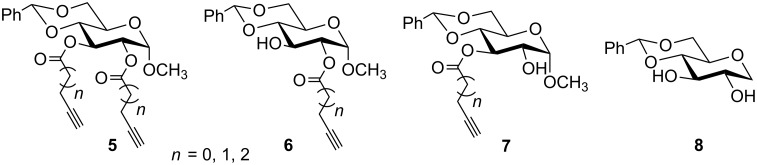
Structures of ester analogs **5–7** and headgroup **8**.

Previously, we have systematically synthesized and studied the self-assembling properties of a series of methyl 4,6-*O*-benzylidene-α-D-glucopyranoside (**1**) derivatives (Figure 1), including esters and carbamates with different functional groups. Several of these compounds proved to be effective gelators for organic solvents and aqueous solutions [34–37]. We found that the structures of the acyl chains of diester **2**, and monoesters **3** and **4,** are important for gelation. This result also indicates that the ester linked derivatives require more specific structures and many substituents are not tolerated. Typically, monoesters with alkynyl groups containing 5–7 carbons (Figure 2, **5–7**) are good gelators for water or aqueous ethanol mixtures. The monoesters presumably form an extended hydrogen bonding array between the ring oxygen and the free hydroxy groups [36].

For these compounds, the main forces that influence gelation include phenyl ring π–π interactions, hydrogen bonding, and hydrophobic interactions of the acyl chains, etc. To further understand the structural influence of the anomeric position of the sugar headgroup on self-assembly, we synthesized analogs using head group **8** [38] (Figure 2), in which the anomeric methoxy group was replaced with a hydrogen atom and contained a similar series of acyl chains to those in compounds **2–4**. These can potentially lead to novel classes of organogelators.

## Results and Discussion

**Scheme 1:**
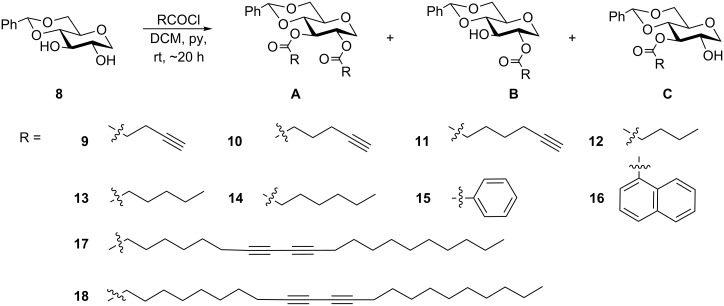
Synthesis of a series of esters **9A–18C**.

In order to better understand the importance of the anomeric substituent of the sugar headgroup for self-assembly, we synthesized a series of esters of the headgroup **8** by a one pot reaction of the acid chloride with the headgroup (Scheme 1). In general, three products were obtained, which could be separated by flash chromatography. Our previous results had shown that esterification of headgroup **1** typically gave the 2-monoester as the major product, but when the headgroup **8** was used, the selectivity of the acylation diminished significantly. The 2- and 3-esters were obtained in similar quantities and, in some cases, the 3-ester was the major product. The difference in the acylation selectivity was possibly because of the configurations of C-1 and C-2 positions. The α-methoxy group allows intramolecular hydrogen bonding to the C-2 hydroxy to take place, which can make the 2-hydroxy group relatively more nucleophilic than the 3 position. In compound **8**, there is no α-methoxy group available for hydrogen bonding to the 2-hydroxy, so the 2- and 3-hydroxy groups were more or less equally nucleophilic [39]. The amount of diester was significantly less than the amounts of the 2- and 3-esters. Diester formation mirrors the synthesis of the diesters of compound **1**.

The selection of the R groups used in this series was based on our previous results [34–36]. We synthesized the terminal acetylenes **9–11**, saturated hydrocarbons **12–14**, aryl derivatives **15**,**16**, and two long chain diacetylene containing glycolipids **17**,**18**. After we obtained these compounds, we then screened them for gelation in several solvents. These results are shown in Table 1.

**Table 1:** The gelation test results of the compounds synthesized^a^.

Compound	Hexane	H_2_O	EtOH	EtOH:H_2_O (1:2)	DMSO:H_2_O (1:2)

**9A**	G 15	I	P	P	P
**10A**	G 20	I	S	P	P
**11A**	P	I	S	P	P
**12A**	S	P	S	P	P
**13A**	S	P	S	S	P
**14A**	S	P	S	P	P
**15A**	I	P	G 5	P	G 5
**16A**	I	P	P	P	G 20
**17A**	S	P	G 7	P	P
**18A**	P	P	G 3	P	P

**9B**	P	I	S	S	G 20
**10B**	P	P	S	P	P
**11B**	P	P	S	P	P
**12B**	P	P	S	P	P
**13B**	P	P	S	P	P
**14B**	P	P	S	P	P
**15B**	I	P	S	G 4	P
**16B**	I	P	S	P	P
**17B**	P	P	S	P	P
**18B**	P	P	S	P	P

**9C**	I	P	S	S	S
**10C**	P	I	S	P	P
**11C**	P	P	S	P	S
**12C**	P	P	S	S	P
**13C**	P	P	S	P	P
**14C**	P	P	S	P	P
**15C**	I	P	S	G 10	G 20
**17C**	P	P	S	P	P
**18C**	P	S	S	P	P

^a^All concentrations are in mg/mL; G, gel at room temperature; the numbers after G are minimum gelation concentrations; P, precipitation; S, soluble at ~20 mg/mL; the ratio of solvents in parenthesis.

From the gelation test results shown in Table 1, quite a few of the diesters were good gelators for the solvents tested. In hexane, only the two short chain terminal acetylene compounds **9A** and **10A** formed gels. Several diesters were effective gelators for ethanol, including the benzoate **15A** and the long chain diacetylene compounds **17A** and **18A**. The two diaryl esters **15A** and **16A** were also able to form gels in aqueous DMSO solution. Compared to the diesters, the monoesters were not as effective as organogelators; only the 2-pentynoate **9B** was able to gelate aqueous DMSO, and the 2-benzoate **15B** was able to form a gel in ethanol. The rest of the 2-monoesters did not gelate any of the other solvents. For the 3-monoesters, compound **15C** was able to form gels in aqueous DMSO and aqueous ethanol, but none of the other esters and solvents formed gels.

**Figure 3:**
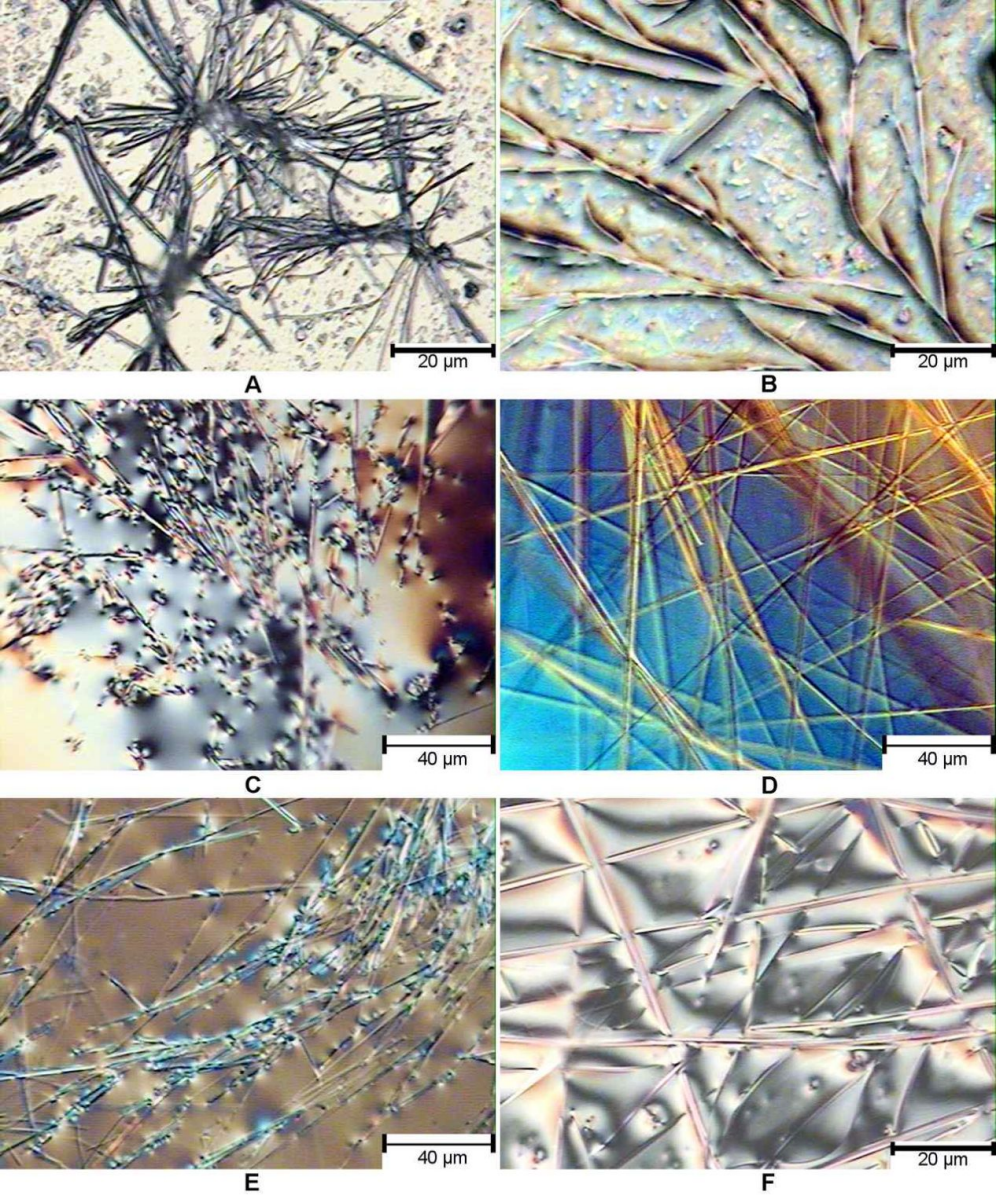
Optical micrographs of the gels formed by compound **9A** in hexane at 15 mg/mL (**A**, **B**), **9B** in DMSO/water (1:2) at 20 mg/mL (**C**, **D**), and **15B** in EtOH/H_2_O (1:2) at 4 mg/mL (**E**, **F**). The images were taken with gels containing solvents (not dried gels).

The morphologies of several gels are shown in Figure 3. The hexane gel of compound **9A** formed fibrous assemblies (Figure 3A, Figure 3B). Compound **9B** showed tubular assemblies (Figure 3C) and more straight cylindrical tube or ribbons (Figure 3D) at different areas. Compound **15B** formed gels more efficiently at 4 mg/mL in the ethanol/water mixture. The morphology of the assembly showed uniform, long and narrow fibers (Figure 3E, Figure 3F).

It is interesting to see that the gelation trends of the diester derivatives of compound **8** are quite different compared to the corresponding compounds derived from headgroup **1**. The gelation of the long chain diacetylene compounds were not affected adversely by changing the headgroup. For the monoesters, the deoxy sugar derivatives are somewhat less effective gelators in comparison to the α-methoxy sugar derivatives. The terminal alkynyl esters were among the most efficient gelators from the ester library derived from compound **1**. The removal of the methoxy group resulted in a sharp reduction in gelation, but these compounds did exhibit some gelation ability. This observation indicated that the α-methoxy group is useful in the formation of a fibrillar network and may or may not be involved in the hydrogen bonding array. However, terminal alkynyl groups did show more promise than saturated hydrocarbons.

**Figure 4:**
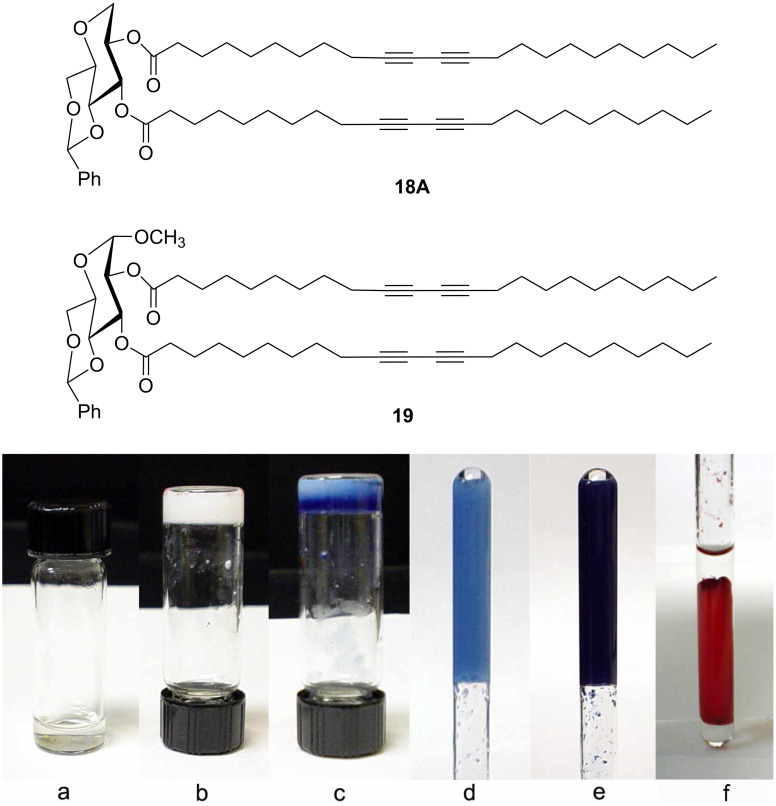
An ethanol gel formed by compound **18A** at <10 mg/mL. a) A clear solution when heated above 70 °C; b) a stable gel after cooling to room temperature; c) a blue gel after illuminating with UV lamp from the top of the vial in b; d) a light blue gel inside quartz tube after UV treatment for 1 min; e) a dark blue gel in quartz tube after UV treatment for 3 min; f) the blue gel in e) turned red after heating.

**Figure 5:**
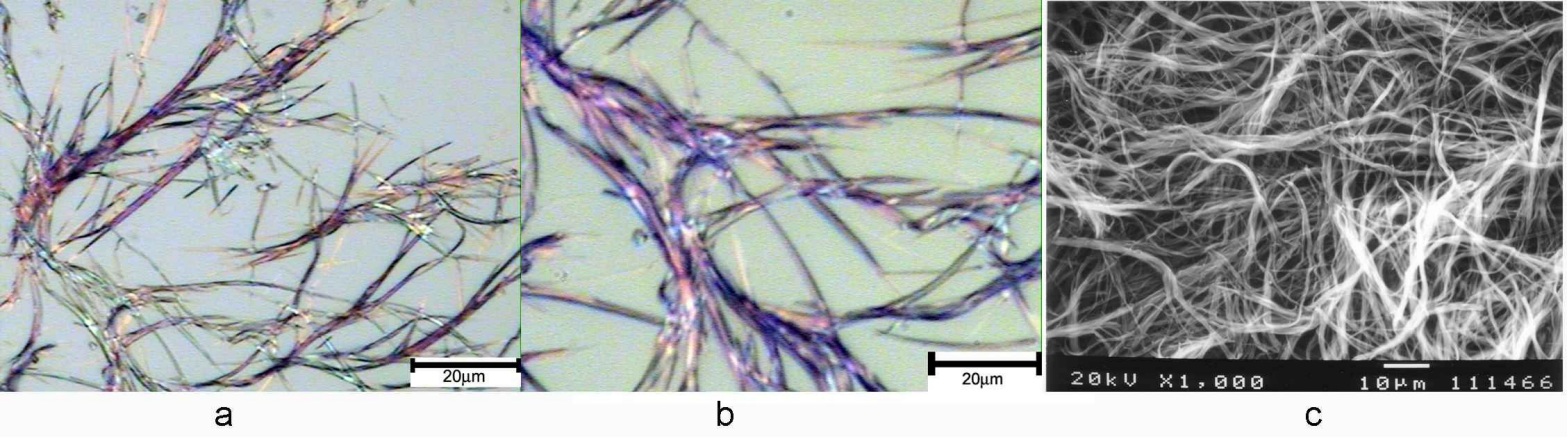
Optical micrographs under bright field (a, b) and scanning electron micrograph (c) of the gel formed by compound **18A** in ethanol after exposure to UV light for 3 min. Blue-purple fibers were observed in (a) and (b), and an entangled fibrous network was observed in (c).

Polydiacetylenes have interesting optical and electronic properties, and diacetylene containing gels may have useful applications as advanced sensing materials [35,40–41]. The morphologies of the self-assembled structures can be retained by cross linking the diacetylene functional groups. Therefore, we have also synthesized and studied several diacetylene containing sugar lipids. Esterification of sugar headgroups with diacetylene containing long chain carboxylic acids can give the desired diacetylene containing lipids. Previously we had used headgroup **1** to synthesize a library of diacetylene containing lipids, and found that many of them are effective gelators [35]. Using the headgroup **8**, we also synthesized six long chain diacetylene containing glycolipids **17A–17C** and **18A–18C**. We found that the diesters are effective gelators for ethanol. Further characterization of the gel formed by compound **18A** in ethanol proved it to be a very efficient gelator, forming gels in ethanol at concentrations lower than 1 wt %/v. In addition, the gels can also be readily polymerized with a 6 W TLC illuminating UV lamp (Figure 4) to give the typically blue colored product. The glass vial is not UV transparent, therefore the polymerization only occurred from the top of the gel (Figure 4c). When a quartz tube was used as the container, the gel turned light blue homogeneously after one min of exposure to UV light (254 nm) (Figure 4d). After three min of UV treatment, it produced a dark blue colored gel (Figure 4e). The blue gel also exhibited interesting color transition properties upon heating (Figure 4f). For a comparison of the two sugar headgroups, the lipid **19** [35] was able gelate ethanol at 7 mg/mL, but it cannot be polymerized as easily with the 6 W UV lamp. This indicates that the two diacetylene chains in **19** are not aligned favorably for polymerization because of the presence of the methoxy group at the anomeric position. The ethanol gels of compound **19** formed long rods or cylindrical tubules, which may require stronger UV energy to polymerize. The optical micrographs of **18A** showed fibrous assemblies composed of long intertwined thin fibers (Figure 5). The topological cross-link of the diacetylenes allowed the fibrous morphologies to be preserved.

**Figure 6:**
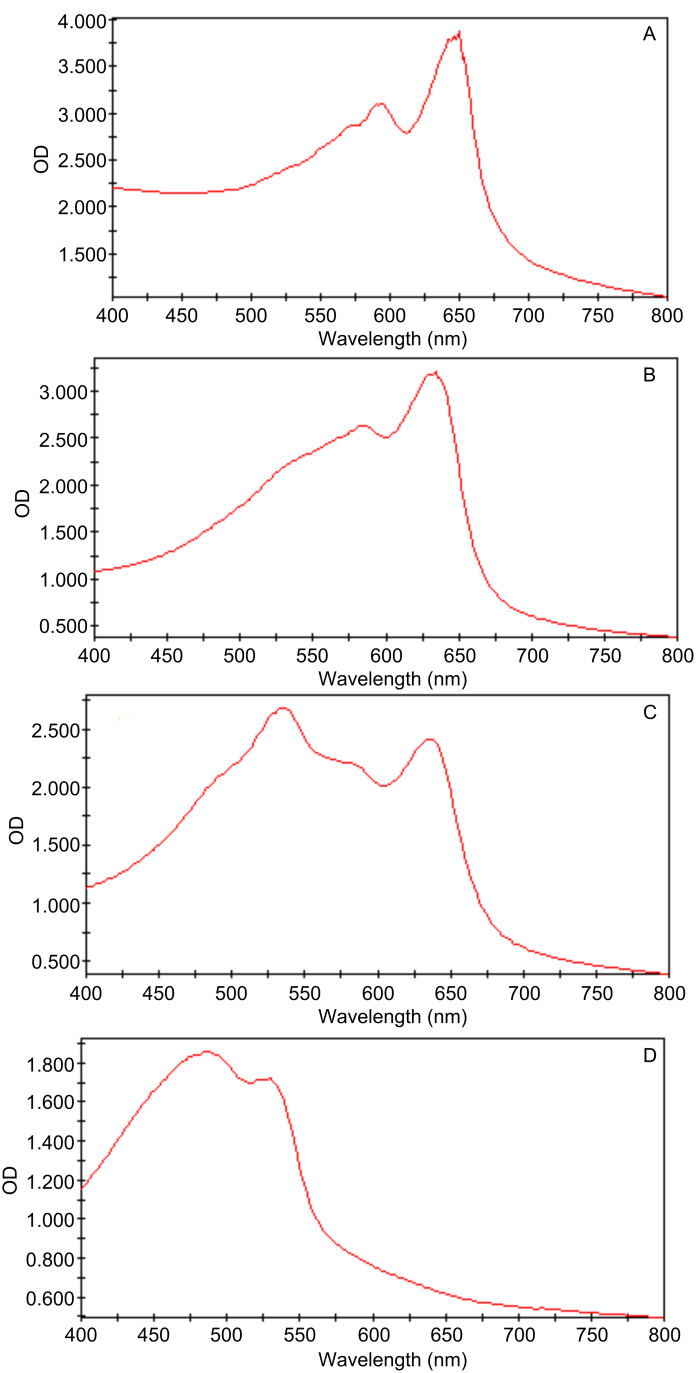
The UV–vis absorption spectra of the polymerized gel formed by compound **18A** in ethanol (10 mg/mL): A, at room temperature after 3 min UV irradiation (λ_max_ = 650 nm); B, at 50 °C ( λ_max_ = 634 nm); C, at 60 °C incubation for 90 min (λ_max_ = 534 nm, other peaks are 582, 636 nm); D, after heating at above 60 °C till complete color change to red (λ_max_ = 486 nm together with 534 nm).

We also carried out UV–vis studies to monitor the color transition of compound **18A** as shown in Figure 6. The gel formed by **18A** in ethanol (10 mg/mL) was treated with a 6 W UV lamp with 254 nm light for 1 min from the top of the uncovered plate. The absorption of the gel was very strong and exceeded the detection limit. The sample was split into two cells, and a small amount of ethanol was added to avoid drying. The UV–vis spectra showed two peaks at 646 nm (λ_max_) and 590 nm, which is in agreement with the observed purple-blue color of the gel. This plate was then treated with the same UV light for another 2 min. The absorption peaks became more intense, and no new signals were observed (Figure 6A). The plate was covered with a matching glass lid and then incubated in an incubator at 35, 40, 50, and 60 °C for 10–15 min. Below 50 °C, there was a small red shift of the λ_max_, which increased with increasing temperature. At 50 °C, the λ_max_ shifted from 650 nm to 634 nm. A short incubation at 60 °C also gave a similar spectrum but a new broad absorption at 530 nm began to appear (Figure 6B). The 530 nm absorption corresponds to the red phase of the gel. After incubating at 60 °C for 90 min, only part of the gel turned red, and the red signal at 530 nm increased significantly as shown in Figure 6C. Upon heating briefly to a higher temperature with a heat gun, the gel turned completely red, and the UV–vis spectrum (Figure 6D) showed two new strong peaks at 486 nm and 528 nm. After cooling the samples, part of the gel turned back to blue whilst the remainder stayed red.

From these observations, we can see that the side chain alignment of compound **18A** is quite favorable in providing long range order for the polymerization of the diacetylene groups. Typically polydiacetylenes exhibit absorptions near 530 nm and 630 nm depending on the side chain structures, and the color transition from blue to red has also been well studied [42–43]. Our gelator **18A** polymerized readily with a TLC UV lamp and the cross linked gels only exhibited absorptions at longer wavelengths. The thermo induced color transition is probably due to an annealing effect of the blue-purple polydiacetylene gels into another stable conformation. The study indicated that the formation of diacetylene gel is an effective method to obtain polydiacetylenes with interesting optical properties.

## Conclusion

We have synthesized a series of 1-deoxy glucose derived esters, and screened their gelation in several solvents. In comparison with the α-methoxy series with the same acyl chains, the deoxy sugar analogs, and the monoesters in particular, are less effective gelators. It appears that the presence of the α-methoxy group assists in the formation of hydrogen bonds among the compounds and with solvents. However, several diester derivatives are effective gelators, and for both the diesters and the monoesters, terminal acetylene and aromatic functional groups seemed to facilitate gelation. Therefore, even though it was found to be less effective than the α-methoxy headgroup **1**, the deoxy sugar headgroup **8** can be used for the preparation of self assembling gelators when the hydrophobic tails are chosen judicially. For instance, long chain diacetylene containing diester derivatives of **8** are effective gelators, and they can be polymerized using a TLC lamp more readily than the corresponding diesters of **1**. The polymerized diacetylene gels were dark blue with long wavelengths absorptions, these compounds may be useful as advanced stimuli responsive materials.

## Experimental

### General methods and materials

**Materials and instrumentation.** General chemicals and reagents were purchased from Aldrich, or VWR. The diacetylene containing fatty acids were purchased from GFS chemicals. Optical microscope images were recorded with an Olympus BX60 microscope and CCD camera. The samples were prepared as thin slices of gels placed on a cleaned glass slide, and the gels were imaged directly under the microscope. NMR spectra were recorded using a 400 MHz Varian NMR spectrometer. High resolution mass spectrometry data were measured on the Q-Tof of the Mass Spectrometry lab at the University of Illinois after the low resolution masses were confirmed. The ionization technique used was ESI (electrospray ionization). Melting points were measured using a Fisher-Jones melting point apparatus.

**Optical microscopy.** Optical micrographs were recorded with an Olympus BX60 microscope and CCD camera. The sample was prepared as a small piece of gel placed on a clean microscope glass slide, and the gel was imaged directly under the microscope. The program used to acquire and store the photos was Corel Photo-Paint 7.

**Scanning electron microscopy.** A piece of the gel was deposited on an aluminum sample holder and allowed to dry in a desiccator. The dried gel sample was coated with a thin layer of platinum (~100–150 Å) by a Denton Vacuum (model Desk II) at a reduced pressure of ~30 mTorr and a current of 45 mA for 60 sec. The sample was analyzed using a JEOL JSM 5410 scanning electron microscope with an EDAX Detecting Unit PV9757/05 ME (Model 204B+, active area = 10 mm^2^).

**Gelation testing.** The compounds were typically tested for gelation in a 1 dram glass vial. The diacetylene containing compounds were tested in brown vials to avoid polymerization. A starting concentration of 20 mg/mL was used. The mixture was heated and sonicated until the sample was fully dissolved. The solution was then allowed to cool to room temperature for 15–20 min. If a gel is formed, then the vial is inverted; if no solvent flows while the gel is inverted, then it is called a stable gel. If the gel falls apart during inversion and by gentle shaking, then it is called an unstable gel or gel-like particulate. If a stable gel is formed, serial dilution is performed until the resulting gel is no longer stable. The concentration prior to formation of the unstable gel was recorded as the minimum gelation concentration (MGC).

**UV–vis spectroscopy.** The UV–vis spectra were recorded on a Bio-Tek PowerWave Microplate Spectrophotometer using a 96-well microplate (Corning’s UV transparent microplate #3635). A sample (about 50–100 μL) of the gel of compound **18A** in ethanol (10 mg/mL) was transferred into a cell of the plate. The plate was covered with its matching lid when not inside the plate holder. Heating was carried out using the internal incubator of the spectrophotometer (temperature range 25–50 °C) and Max 4000 incubator (temperature range 25–60 °C) from Barnstead.

### General procedure for the synthesis of ester derivatives of compound **8**

The three ester derivatives **A**, **B**, and **C** were synthesized in a one pot reaction by reacting the corresponding acid chloride (1.2 equiv) with headgroup **8** (1.0 equiv). Compound **8** was prepared according to literature procedure [38]. If the acid chloride was not commercially available, it was prepared by converting the corresponding terminal alkynyl carboxylic acid (1.2 equiv) to the acyl chloride using excess oxalyl chloride [35]. After confirming complete conversion to the acyl chloride, hexane was used to co-distill and remove the excess oxalyl chloride. The acyl chloride was added to compound **8** (1 equiv) in DCM and 4 equiv of pyridine. The reaction mixture was left stirring under an anhydrous atmosphere for 14–20 h. The crude product was concentrated, extracted with DCM, washed with water, then brine, and the combined organic phase dried over anhydrous sodium sulfate. The crude product was isolated and purified using a gradient solvent system (hexanes and acetone), starting with 5% acetone. All yields reported are pure isolated yields, and were calculated based on compound **8**; the yields were not optimized. The following sections include the characterization data for three compounds, the rest are shown in the Supporting Information section.

### Synthesis of 4-pentynoates **9A**, **9B**, and **9C**

**Compound 9A.** This product was isolated as a white crystalline solid in 9.1% yield. M.p. 97–98 °C. ^1^H NMR (400 MHz, CDCl_3_) δ (ppm) 7.39–7.46 (m, 2H), 7.29–7.37 (m, 3H), 5.49 (s, 1H), 5.37 (t, 1H, *J* = 9.5 Hz), 5.08 (ddd~dt, 1H, *J* = 5.9, 9.5, 10.3 Hz), 4.33 (dd, 1H, *J* = 5.1, 10.3 Hz), 4.13 (dd, 1H, *J* = 5.9, 11.4 Hz), 3.71 (t, 1H, *J* = 10.3 Hz), 3.64 (t, 1H, *J* = 9.5 Hz), 3.46 (dt, 1H, *J* = 5.1, 9.5 Hz), 3.40 (t, 1H, *J* = 11.0 Hz), 2.51–2.59 (m, 4H), 2.43–2.50 (m, 4H), 1.99 (t, 1H, *J* = 2.6 Hz), 1.89 (t, 1H, *J* = 2.6 Hz). ^13^C NMR (100 MHz, CDCl_3_) δ (ppm) 170.8, 170.7, 136.8, 129.0, 128.1, 126.1, 101.4, 82.2, 82.0, 78.6, 72.5, 71.4, 69.8, 69.3, 68.5, 67.3, 33.2, 33.1, 14.3, 14.2. HRMS ESI calcd for C_23_H_24_O_7_Na [M + Na^+^] 435.1420, found 435.1406.

**Compound 9B**. This product was isolated as a white crystalline solid in 14.6% yield. M.p. 100–101 °C. ^1^H NMR (400 MHz, CDCl_3_) δ (ppm) 7.46–7.53 (m, 2H), 7.33–7.42 (m, 3H), 5.51 (s, 1H), 4.93 (ddd~dt, 1H, *J* = 5.9, 9.5, 10.3 Hz), 4.30 (dd, 1H, *J* = 4.8, 10.6 Hz), 4.09 (dd, 1H, *J* = 5.9, 11.0 Hz), 3.85 (t, 1H, *J* = 9.2 Hz), 3.67 (t, 1H, *J* = 10.3 Hz), 3.49 (t, 1H, *J* = 9.2 Hz), 3.35 (dt, 1H, *J* = 5.1, 9.9 Hz), 3.26 (dd~t, 1H, *J* = 10.6, 11.0 Hz), 2.54–2.64 (m, 2H), 2.45–2.54 (m, 2H), 2.01 (t, 1H, *J* = 2.6 Hz). ^13^C NMR (100 MHz, CDCl_3_) δ (ppm) 171.1, 136.8, 129.2, 128.2, 126.2, 101.8, 82.2, 81.0, 72.4, 71.9, 70.9, 69.3, 68.5, 67.0, 33.1, 14.3. HRMS ESI calcd for C_18_H_20_O_6_Na [M + Na^+^] 355.1158, found 355.1166.

**Compound 9C**. This product was isolated as a white crystalline solid in 23.0% yield. M.p. 148–150 °C. ^1^H NMR (400 MHz, CDCl_3_) δ (ppm) 7.40–7.47 (m, 2H), 7.28–7.36 (m, 3H), 5.46 (s, 1H), 5.16 (dd~t, 1H, *J* = 9.2, 9.5 Hz), 4.30 (dd, 1H, *J* = 5.1, 10.6 Hz), 4.07 (dd, 1H, *J* = 5.9, 11.4 Hz), 3.86 (m, 1H), 3.67 (dd~t, 1H, *J* = 10.1 Hz), 3.56 (dd~t, 1H, *J* = 9.3 Hz, 1H), 3.34–3.47 (m, 2H), 2.54–2.64 (m, 2H), 2.42–2.52 (m, 2H), 1.88 (m, 1H). ^13^C NMR (100 MHz, CDCl_3_) δ (ppm) 172.0, 136.9, 128.9, 128.1, 126.0, 101.2, 82.3, 78.7, 76.6, 71.2, 70.6, 69.14, 69.09, 68.7, 33.3, 14.3. HRMS calcd for C_18_H_20_O_7_Na [M + Na^+^] 355.1158, found 355.1147.

## Supporting Information

File 1 Yields and characterization data for compounds **10A**–**10C** to **18A**–**18C**.

File 2 ^1^H and ^13^C NMR spectra of compounds **9A–18C**.
