# Supplementary material for: Synthesis and self-assembly of 1-deoxyglucose derivatives as low molecular weight organogelators
Source: Beilstein J Org Chem. 2011 Feb 21;7:234–42. doi: 10.3762/bjoc.7.31 (PMC3062983; doi:10.3762/bjoc.7.31)
Supplement: File 1 — Yields and characterization data for compounds 10A–10C to 18A–18C. [file Beilstein_J_Org_Chem-07-234-s001.pdf]

Supporting Information

for

Synthesis and self-assembly of 1-deoxyglucose derivatives as low molecular weight organogelators

Guijun Wang*[1], Hao Yang[1], Sherwin Cheuk[1] and Sherman Coleman[2]

Address: [1]Department of Chemistry, University of New Orleans, New Orleans, LA 70148, Phone: 504 280-1258, Fax: 504 280-6860 and [2]Dillard University, 2601 Gentilly Boulevard, New Orleans, Louisiana 70122

Email: Guijun Wang - gwang2@uno.edu

*Corresponding author

Yields and characterization data for compounds 10A–10C to 18A–18C

Synthesis of 5-hexynoates 10A, 10B, and 10C

Compound 10A. This product was isolated as a white crystalline solid in 4.5% yield. M.p. 88–90 °C. ^{1}H NMR (400 MHz, $CDCl_3$) δ (ppm) 7.40–7.46 (m, 2H), 7.31–7.38 (m, 3H), 5.50 (s, 1H), 5.36 (t, 1H, *J* = 9.5 Hz), 5.05 (m, 1H), 4.34 (dd, 1H, *J* = 4.9, 10.5 Hz), 4.13 (dd, 1H, *J* = 5.9, 11.0 Hz), 3.71 (t, 1H, *J* = 10.3 Hz), 3.64 (t, 1H, *J* = 9.5 Hz), 3.47 (dd~t, 1H, *J* = 4.8, 9.5, 9.9 Hz), 3.39 (t, 1H, *J* = 10.8 Hz), 2.41–2.50 (m, 4H), 2.24 (dt, 2H, *J* = 2.6, 6.9 Hz), 2.21 (dt, 2H, *J* = 2.6, 6.9 Hz), 1.98 (t, 1H, *J* = 2.6), 1.94 (t, 1H, *J* = 2.6), 1.81 (m, 4H). ^{13}C NMR (100 MHz, $CDCl_3$) δ (ppm) 172.2, 172.1, 136.8, 129.1, 128.2, 126.1, 101.4, 83.0, 82.9, 78.8, 72.3, 71.4, 69.5, 69.4, 69.2, 68.6, 67.4, 32.7, 32.6, 23.8, 23.3, 17.7, 17.6. HRMS Calcd for $C_{25}H_{28}O_7Na$ $[M + Na]^+$ 463.1733, found 463.1743.

Compound 10B. This product was isolated as a white crystalline solid in 16.3% yield. M.p. 75–77 °C. ^{1}H NMR (400 MHz, $CDCl_3$) δ (ppm) 7.46–7.53 (m, 2H), 7.33–7.43 (m, 3H), 5.54 (s, 1H), 4.95 (ddd, 1H, *J* = 5.9, 9.2, 10.6 Hz), 4.33 (dd, 1H, *J* = 5.0, 10.5 Hz), 4.11 (dd, 1H, *J* = 5.9, 11.4 Hz), 3.89 (t, 1H, *J* = 9.2 Hz), 3.70 (t, 1H, *J* = 10.3 Hz), 3.52 (t, 1H, *J* = 9.2 Hz), 3.38 (dt, 1H, *J* = 5.0, 9.7 Hz), 3.29 (t, 1H, *J* = 10.8 Hz), 2.51 (m, 2H), 2.27 (dt, 2H, *J* = 2.6, 7.0 Hz), 1.98 (t, 1H, *J* = 2.6 Hz), 1.86 (p, 2H, *J* = 7.0 Hz). ^{13}C NMR (100 MHz, $CDCl_3$) δ (ppm) 172.6, 136.9, 129.3, 128.3, 126.2, 101.9, 83.1, 81.1, 72.7, 71.6, 71.0, 69.3, 68.6, 67.2, 32.7, 23.4, 17.7. HRMS Calcd $C_{19}H_{22}O_7Na$ $[M + Na]^+$ 369.1314, found 369.1319.

Compound 10C. This product was isolated as a white crystalline solid in 19.5% yield. M.p. 102–103 °C. ^{1}H NMR (400 MHz, $CDCl_3$) δ (ppm) 7.43–7.47 (m, 2H), 7.33–7.39 (m, 3H), 5.51

(s, 1H), 5.04 (t, 1H, *J* = 9.2 Hz), 4.35 (dd, 1H, *J* = 4.8, 10.6 Hz), 4.11 (dd, 1H, *J* = 5.9, 11.4 Hz), 3.85 (m, 1Hz), 3.70 (t, 1H, *J* = 10.3 Hz), 3.61 (t, 1H, *J* = 9.3 Hz), 3.44 (dt, 1H, *J* = 5.1, 9.9 Hz), 3.32 (t, 1H, *J* = 11.0 Hz), 2.91 (d, 1H, *J* = 5.1 Hz), 2.56 (t, 1H, *J* = 7.3 Hz), 2.25 (dt, 2H, *J* = 2.6, 7.0 Hz), 1.95 (t, 1H, *J* = 2.6 Hz), 1.87 (p, 2H, *J* = 7.0 Hz). ^{13}C NMR (100 MHz, $CDCl_3$) δ (ppm) 173.7, 136.8, 128.9, 128.0, 125.9, 101.2, 83.1, 78.5, 76.1, 71.1, 70.4, 69.2, 69.1, 68.5, 32.8, 23.5, 17.4. HRMS Calcd for $C_{19}H_{22}O_7Na$ $[M + Na]^+$ 369.1314, found 369.1308.

Synthesis of 6-heptynoates 11A, 11B, and 11C

Compound 11A. This product was isolated as an oil in 3.3% yield. ^{1}H NMR (400 MHz, $CDCl_3$) δ (ppm) 7.39–7.46 (m, 2H), 7.30–7.38 (m, 3H), 5.50 (s, 1H), 5.36 (t, 1H, *J* = 9.5 Hz), 5.05 (ddd~dt, 1H, *J* = 5.9, 9.7, 10.3 Hz), 4.34 (dd, 1H, *J* = 4.9, 10.3 Hz), 4.12 (dd, 1H, *J* = 5.9, 11.0 Hz), 3.71 (t, 1H, *J* = 10.3 Hz), 3.63 (t, 1H, *J* = 9.5 Hz), 3.47 (dt, 1H, *J* = 4.8, 9.5 Hz), 3.39 (t, 1H, *J* = 10.8 Hz), 2.28–2.39 (m, 4H), 2.20 (dt, 2H, *J* = 2.6, 7.0 Hz), 2.11 (dt, 2H, *J* = 2.6, 7.0 Hz), 1.96 (t, 1H, *J* = 2.6 Hz), 1.92 (t, 1H, *J* = 2.6 Hz), 1.71 (m, 4H), 1.52 (m, 4H). ^{13}C NMR (100 MHz, $CDCl_3$) δ (ppm) 172.5, 172.4, 136.8, 129.1, 128.2, 126.1, 101.4, 83.8, 83.7, 78.8, 72.2, 71.4, 69.4, 68.7, 68.62, 68.57, 67.5, 33.7, 33.5, 27.7, 27.5, 24.0, 23.8, 18.1, 18.0. HRMS Calcd for $C_{27}H_{33}O_7$ $[M + H]^+$ 469.2226, found 469.2219.

Compound 11B. This product was isolated as a white crystalline solid in 21.5% yield. M.p. 118–119 °C. ^{1}H NMR (400 MHz, $CDCl_3$) δ (ppm) ^{1}H NMR (400 MHz, CDCl3) δ 7.45–7.53 (m, 2H), 7.33–7.41 (m, 3H), 5.55 (s, 1H), 4.93 (ddd~dt, 1H, *J* = 5.9, 9.5, 10.3 Hz), 4.34 (dd, 1H, *J* = 4.9, 10.4 Hz), 4.12 (dd, 1H, *J* = 5.9, 11.2 Hz), 3.88 (t, 1H, *J* = 9.2 Hz), 3.69 (t, 1H, *J* = 10.3 Hz), 3.52 (t, 1H, *J* = 9.3 Hz), 3.40 (dt, 1H, *J* = 5.1, 9.7 Hz), 3.27 (t, 1H, *J* = 10.8 Hz), 2.30–2.45 (m,

2H), 2.15–2.25 (m, 2H), 1.95 (m, 1H), 1.68–1.80 (m, 2H), 1.57 (m, 2H). ^{13}C NMR (100 MHz, $CDCl_3$) δ (ppm) 172.8, 136.8, 129.1, 128.2, 126.1, 101.7, 83.8, 81.0, 72.2, 71.4, 70.9, 68.6, 68.4, 67.1, 33.3, 27.4, 23.6, 17.9. HRMS Ccalcd for $C_{20}H_{24}O_6$ Na [M + Na] $^+$ 383.1471, found 383.1480.

Compound 11C. This product was isolated as a white crystalline solid in 20.1% yield. M.p. 79–81 °C. ^{1}H NMR (400 MHz, $CDCl_3$) δ (ppm) ^{1}H NMR (400 MHz, $CDCl_3$) δ 7.42–7.47 (m, 2H), 7.31–7.40 (m, 3H), 5.49 (s, 1H), 5.08 (t, 1H, *J* = 9.2 Hz), 4.31 (dd, 1H, *J* = 4.9, 10.4 Hz), 4.05 (dd, 1H, *J* = 5.9, 11.4 Hz), 3.79 (dt, 1H, *J* = 5.9, 10.3 Hz), 3.68 (t, 1H, *J* = 10.3 Hz), 3.57 (t, 1H, *J* = 9.5 Hz), 3.43 (dt, 1H, *J* = 4.8, 9.5 Hz), 3.34 (t, 1H, *J* = 11.0 Hz), 3.20 (sb, 1H), 2.43 (t, 2H, *J* = 7.3 Hz), 2.12 (dt, 2H, *J* = 2.6, 6.9 Hz), 1.94 (t, 1H, *J* = 2.6 Hz), 1.76 (m, 2H), 1.52 (m, 2H). ^{13}C NMR (100 MHz, $CDCl_3$) δ 174.6, 136.8, 129.0, 128.1, 126.0, 101.3, 83.8, 78.5, 76.5, 71.1, 70.4, 69.6, 68.7, 68.6, 33.7, 27.3, 23.8, 17.9. HRMS Calcd for $C_{20}H_{24}O_6$Na [M + Na]$^+$ 383.1471, found 383.1487.

Synthesis of pentanoates 12A, 12B and 12C

Compound 12A. This product was isolated as a white crystalline solid in 18.0% yield. M.p. 71–72 °C ^{1}H NMR (400 MHz, $CDCl_3$) δ (ppm) 7.40–7.46 (m, 2H), 7.32–7.37 (m, 3H), 5.50 (s, 1H), 5.36 (t, 1H, *J* = 9.5 Hz), 5.05 (m, 1H), 4.34 (dd, 1H, *J* = 4.9, 10.5 Hz), 4.12 (dd, 1H, *J* = 5.9, 11.0 Hz), 3.72 (t, 1H, *J* = 10.3 Hz), 3.63 (t, 1H, *J* = 9.5 Hz), 3.47 (dt, 1H, *J* = 4.8, 9.5, 9.9 Hz), 3.39 (t, 1H, *J* = 10.8 Hz), 2.26–2.34 (m, 4H), 1.52–1.64 (m, 4H), 1.23–1.38 (m, 4H), 0.90 (t, 3H, *J* = 7.3 Hz), 0.94 (t, 3H, *J* = 7.3 Hz). ^{13}C NMR (100 MHz, $CDCl_3$) δ (ppm) 172.8, 136.9, 129.0, 128.2,

126.0, 101.3, 78.9, 72.0, 71.4 69.3, 68.6, 67.5, 33.9, 33.7, 27.0, 26.8, 22.1, 22.0, 13.6. HRMS Calcd for $C_{23}H_{33}O_7$ $[M + H]^+$ 420.2226, found 421.2217.

Compound 12B. This product was isolated as a white crystalline solid in 31.5% yield. M.p. 107–109 °C ^1H NMR (400 MHz, $CDCl_3$) δ (ppm) 7.47–7.52 (m, 2H), 7.35–7.41 (m, 3H), 5.55 (s, 1H), 4.95 (ddd, 1H, *J* = 5.9, 9.2, 10.6 Hz), 4.33 (dd, 1H, *J* = 5.1, 10.6 Hz), 4.12 (dd, 1H, *J* = 5.9, 11.0 Hz), 3.92 (t, 1H, *J* = 9.2 Hz), 3.71 (t, 1H, *J* = 10.3 Hz), 3.55 (t, 1H, *J* = 9.2 Hz), 3.40 (dt, 1H, *J* = 4.8, 9.5 Hz), 3.30 (t, 1H, *J* = 10.8 Hz), 2.62 (s, 1H), 2.37 (m, 2H), 1.62 (m, 2H), 1.36 (hex, 2H, *J* = 7.3 Hz), 0.92 (t, 3H, *J* = 7.3 Hz). ^{13}C NMR (100 MHz, $CDCl_3$) δ (ppm) 173.4, 136.9, 129.3, 128.4, 126.3, 101.9, 81.2, 72.9, 71.5, 71.0, 68.7, 67.3, 33.8, 26.9, 22.1, 13.7. HRMS Calcd for $C_{18}H_{25}O_6$ $[M + H]^+$ 337.1651, found 337.1644.

Compound 12C. This product was isolated as a white crystalline solid in 23.3% yield. M.p. 72–74 °C. ^1H NMR (400 MHz, $CDCl_3$) δ (ppm) 7.42–7.48 (m, 2H), 7.33–7.38 (m, 3H), 5.51 (s, 1H), 5.00 (t, 1H, *J* = 9.2 Hz), 4.35 (dd, 1H, *J* = 4.8, 10.6 Hz), 4.11 (dd, 1H, *J* = 5.9, 11.4 Hz), 3.83 (m, 1H), 3.70 (t, 1H, *J* = 10.3 Hz), 3.61 (t, 1H, *J* = 9.3 Hz), 3.44 (dt, 1H, *J* = 4.8, 9.9 Hz), 3.39 (t, 1H, *J* = 11.0 Hz), 2.95 (d, 1H, *J* = 4.4 Hz), 2.41 (t, 2H, *J* = 7.5 Hz), 1.63 (pentet, 2H, *J* = 7.5 Hz), 1.34 (hex, 2H, *J* = 7.5 Hz), 0.87 (t, 3H, *J* = 7.5 Hz). ^{13}C NMR (100 MHz, $CDCl_3$) δ (ppm) 175.7, 136.9, 129.1, 128.2, 126.1, 101.4, 78.4, 77.1, 71.4, 70.6, 70.1, 68.8, 34.1, 27.0, 22.1, 13.6. HRMS Calcd for $C_{18}H_{25}O_6$ $[M + H]^+$ 337.1651, found 337.1646.

Synthesis of hexanoates 13A, 13B and 13C

Compound 13A. This product was isolated as a white crystalline solid in 12.4% yield. M.p. 37–38 °C. ^{1}H NMR (400 MHz, $CDCl_3$) δ (ppm) 7.40–7.46 (m, 2H), 7.32–7.38 (m, 3H), 5.50 (s, 1H), 5.36 (t, 1H, *J* = 9.5 Hz), 5.05 (ddd~dt, 1H, *J* = 5.9, 9.5, 10.3 Hz), 4.34 (dd, 1H, *J* = 4.9, 10.5 Hz), 4.12 (dd, 1H, *J* = 5.9, 11.2 Hz), 3.72 (t, 1H, *J* = 10.3 Hz), 3.63 (t, 1H, *J* = 9.5 Hz), 3.47 (dt, 1H, *J* = 5.1, 9.9 Hz), 3.39 (t, 1H, *J* = 10.8 Hz), 2.25–2.37 (m, 4H), 1.53–1.64 (m, 4H), 1.20–1.36 (m, 8H), 0.89 (t, 3H, *J* = 7.0 Hz), 0.82 (t, 3H, *J* = 7.0 Hz). ^{13}C NMR (100 MHz, $CDCl_3$) δ (ppm) 172.8, 136.9, 129.0, 128.2, 126.0, 101.4, 78.9, 72.0, 71.5, 69.4, 68.6, 67.5, 34.3, 34.0, 31.15, 31.1, 24.7, 24.5, 22.2, 13.9, 13.8. HRMS Calcd for $C_{25}H_{37}O_7$ $[M + H]^+$ 449.2539, found 449.2538.

Compound 13B. This product was isolated as a white crystalline solid in 31.7% yield. M.p. 112–114 °C. ^{1}H NMR (400 MHz, $CDCl_3$) δ (ppm) 7.46–7.53 (m, 2H), 7.35–7.42 (m, 3H), 5.55 (s, 1H), 4.95 (m, 1H), 4.33 (dd, 1H, *J* = 4.8, 10.6 Hz), 4.12 (dd, 1H, *J* = 5.7, 11.2 Hz), 3.91 (t, 1H, *J* = 9.2 Hz), 3.71 (t, 1H, *J* = 10.3 Hz), 3.54 (t, 1H, *J* = 9.3 Hz), 3.40 (dt, 1H, *J* = 4.8, 9.5 Hz), 3.30 (t, 1H, *J* = 10.8 Hz), 2.64 (s, 1H), 2.36 (m, 2H), 1.64 (p, 2H, *J* = 7.3 Hz), 1.31 (m, 4H), 0.90 (t, 3H, *J* = 6.8 Hz). ^{13}C NMR (100 MHz, $CDCl_3$) δ (ppm) 173.4, 136.9, 129.3, 128.3, 126.2, 101.9, 81.2, 72.8, 71.5, 71.0, 68.7, 67.3, 34.1, 31.2, 24.5, 22.3, 13.9. HRMS Calcd for $C_{19}H_{27}O_6$ $[M + H]^+$ 351.1808, found 351.1801.

Compound 13C. This product was isolated as a white crystalline solid in 19.5% yield. M.p. 113–114 °C. 11^{1}H NMR (400 MHz, $CDCl_3$) δ (ppm) 7.42–7.48 (m, 2H), 7.33–7.39 (m, 3H), 5.51 (s, 1H), 5.01 (t, 1H, *J* = 9.2 Hz), 4.35 (dd, 1H, *J* = 4.9, 10.5 Hz), 4.11 (dd, 1H, *J* = 5.7, 11.5

Hz), 3.83 (m, 1H), 3.70 (t, 1H, *J* = 10.3 Hz), 3.61 (t, 1H, *J* = 9.5 Hz), 3.44 (dt, 1H, *J* = 4.8, 9.9 Hz), 3.39 (t, 1H, *J* = 10.8 Hz), 2.98 (sb, 1H), 2.40 (t, 2H, *J* = 7.3 Hz), 1.65 (m, 2H), 1.22–1.36 (m, 4H), 0.84 (t, 3H, *J* = 7.0 Hz). ^{13}C NMR (100 MHz, $CDCl_3$) δ (ppm) 175.7, 137.0, 129.1, 128.2, 126.1, 101.4, 78.4, 77.1, 71.4, 70.6, 70.1, 68.8, 34.3, 31.1, 24.7, 22.2, 13.8 HRMS Calcd for $C_{19}H_{27}O_6$ $[M + H]^+$ 351.1808, found 351.1801.

Synthesis of heptanoates 14A, 14B and 14C

Compound 14A. This product was isolated as a white crystalline solid in 38.2% yield. M.p. 49–50 °C. ^{1}H NMR (400 MHz, $CDCl_3$) δ (ppm) 7.40–7.46 (m, 2H), 7.32–7.38 (m, 3H), 5.49 (s, 1H), 5.36 (t, 1H, *J* = 9.5 Hz), 5.05 (ddd, 1H, *J* = 5.9, 9.5, 10.3 Hz), 4.34 (dd, 1H, *J* = 4.9, 10.5 Hz), 4.12 (dd, 1H, *J* = 5.9, 11.0 Hz), 3.71 (t, 1H, *J* = 10.3 Hz), 3.63 (t, 1H, *J* = 9.5 Hz), 3.47 (dt, 1H, *J* = 4.8, 9.9 Hz), 3.38 (t, 1H, *J* = 11.0 Hz), 2.24–2.37 (m, 4H), 1.53–1.64 (m, 4H), 1.17–1.36 (m, 12H), 0.88 (t, 3H, *J* = 7.0 Hz), 0.84 (t, 3H, *J* = 7.0 Hz). ^{13}C NMR (100 MHz, $CDCl_3$) δ (ppm) 172.79, 172.76, 136.9, 128.9, 128.1, 126.0, 101.3, 78.8, 72.0, 71.4, 69.3, 68.6, 67.4, 34.2, 34.0, 31.4, 31.3, 28.64, 28.60, 24.9, 24.7, 22.4, 22.3, 14.0. HRMS Calcd for $C_{27}H_{41}O_7$ $[M + H]^+$ 477.2852, found 477.2847.

Compound 14B. This product was isolated as a white crystalline solid in 22.9% yield. M.p. 102–104 °C. ^{1}H NMR (400 MHz, $CDCl_3$) δ (ppm) 7.46–7.53 (m, 2H), 7.35–7.42 (m, 3H), 5.55 (s, 1H), 4.95 (ddd, 1H, *J* = 5.9, 9.2, 10.3 Hz), 4.34 (dd, 1H, *J* = 5.1, 10.6 Hz), 4.12 (dd, 1H, *J* = 5.9, 11.0 Hz), 3.92 (t, 1H, *J* = 9.2 Hz), 3.71 (t, 1H, *J* = 10.3 Hz), 3.55 (t, 1H, *J* = 9.2 Hz), 3.40 (dt, 1H, *J* = 5.0, 9.7 Hz), 3.30 (t, 1H, *J* = 10.8 Hz), 2.65 (sb, 1H), 2.36 (m, 2H), 1.63 (p, 2H, *J* = 7.3 Hz), 1.24–1.39 (m, 6H), 0.88 (t, 3H, *J* = 6.8 Hz). ^{13}C NMR (100 MHz, $CDCl_3$) δ (ppm)

173.4, 136.9, 129.3, 128.3, 126.2, 101.9, 81.2, 72.8, 71.5, 71.0, 68.7, 67.3, 34.1, 31.4, 28.7, 24.8, 22.4, 14.0; HRMS Calcd for $C_{20}H_{29}O_6$ $[M + H]^+$ 365.1964, found 365.1958.

Compound 14C. This product was isolated as a white crystalline solid in 20.8% yield. M.p. 89–91 °C. ^{1}H NMR (400 MHz, $CDCl_3$) δ (ppm) 7.42–7.49 (m, 2H), 7.32–7.40 (m, 3H), 5.51 (s, 1H), 5.01 (t, 1H, *J* = 9.2 Hz), 4.35 (dd, 1H, *J* = 4.9, 10.6 Hz), 4.11 (dd, 1H, *J* = 5.9, 11.4 Hz), 3.83 (m, 1H), 3.71 (t, 1H, *J* = 10.3 Hz), 3.61 (t, 1H, *J* = 9.3 Hz), 3.44 (dt, 1H, *J* = 4.9, 9.9 Hz), 3.39 (t, 1H, *J* = 11.0 Hz), 2.97 (sb, 1H), 2.41 (t, 2H, *J* = 7.5 Hz), 1.64 (p, 2H, *J* = 7.3 Hz), 1.15–1.36 (m, 6H), 0.84 (t, 3H, *J* = 6.6 Hz). ^{13}C NMR (100 MHz, $CDCl_3$) δ (ppm) 175.7, 137.0, 129.1, 128.2, 126.1, 101.4, 78.4, 77.1, 71.4, 70.6, 70.1, 68.8, 34.4, 31.4, 28.6, 25.0, 22.4, 14.0; HRMS Calcd for $C_{20}H_{29}O_6$ $[M + H]^+$ 365.1964, found 365.1953.

Synthesis of benzoate derivatives 15A, 15B and 15C[34]

Compound 15A. This product was isolated as a white crystalline solid in 7.8% yield. M.p. 162–163 °C. ^{1}H NMR (400 MHz, $CDCl_3$) δ (ppm) 7.94–8.03 (m, 4H), 7.47–7.55 (m, 2H), 7.35–7.45 (m, 6H), 7.29–7.34 (m, 3H), 5.83 (t, 1H, *J* = 9.5 Hz), 5.56 (s, 1H), 5.40 (m, 1H), 4.42 (dd, 1H, *J* = 4.9, 10.5 Hz), 4.38 (dt, 1H, *J* = 4.8, 9.7 Hz), 3.89 (t, 1H, *J* = 9.5 Hz), 3.81 (t, 1H, *J* = 10.3 Hz), 3.61 (t, 1H, *J* = 9.3 Hz), 3.63 (dt, 1H, *J* = 4.9, 9.9 Hz), 3.58 (t, 1H, *J* = 10.8 Hz). ^{13}C NMR (100 MHz, $CDCl_3$) δ (ppm) 165.8, 165.7, 136.8, 133.4, 133.1, 129.8, 129.76, 129.6, 129.0, 128.5, 128.3, 128.2, 126.1, 101.5, 79.1, 72.7, 71.7, 70.5, 68.7, 67.7; HRMS Calcd for $C_{27}H_{25}O_7$ $[M + H]^+$ 460.1600, found 461.1589.

Compound 15B[34]**.** This product was isolated as a white crystalline solid in 18.7% yield. M.p. 126–127 °C. ^{1}H NMR (400 MHz, $CDCl_3$) ^{1}H NMR ($CDCl_3$, 400MHz) δ 8.05 (d, 2H, J = 7.1 Hz), 7.60–7.37 (m, 8H), 5.57 (s, 1H), 5.19 (ddd, 1H, J = 10.6, 9.2, 5.9 Hz), 4.36 (dd, 1H, J = 10.3, 5.0 Hz), 4.26 (dd, 1H, J = 11.0, 5.9 Hz), 4.08 (t, 1H, J = 9.2 Hz), 3.75 (t, 1H, J = 10.3 Hz), 3.61 (dd~t, 1H, J = 9.5, 9.2 Hz), 3.45 (m, 2H); ^{13}C NMR ($CDCl_3$, 100MHz) δ 166.0, 136.9, 133.3, 129.7, 129.3, 129.2, 128.4, 128.3, 126.3, 101.8, 81.1, 72.6, 72.1, 71.0, 68.5, 67.2.

Compound 15C. This product was isolated as a white crystalline solid in 32.4% yield. M.p. 155–156 °C. ^{1}H NMR (400 MHz, $CDCl_3$) δ (ppm) 8.08 (d, 2H, J = 7.7 Hz), 7.58 (m, 1H), 7.41–7.50 (m, 4H), 7.30–7.37 (m, 3H), 5.56 (s, 1H), 5.30 (t, 1H, J = 9.5 Hz), 4.38 (dd, 1H, J = 4.9, 10.5 Hz), 4.15 (dd, 1H, J = 5.9, 11.4 Hz), 3.98 (ddd, 1H, J = 5.9, 9.2, 10.5 Hz), 3.72–3.81 (m, 2H), 3.51 (dt, 1H, J = 4.9, 9.5 Hz), 3.46 (t, 1H, J = 11.0 Hz); ^{13}C NMR (100 MHz, $CDCl_3$) δ (ppm) 167.9, 136.9, 133.5, 129.9, 129.4, 129.0, 128.4, 128.2, 126.0, 101.4, 78.6, 77.8, 71.4, 70.6, 70.0, 68.8. HRMS Calcd for $C_{20}H_{21}O_6$ $[M + H]^+$ 357.1338, found 357.1329.

Synthesis of naphthoates 16A and 16B

Compound 16A. This product was isolated as a white crystalline solid in 32.5% yield. M.p. 135–137 °C. ^{1}H NMR (400 MHz, $CDCl_3$) δ (ppm) 8.87 (d, 1H, J = 8.4 Hz), 8.68 (d, 1H, J = 8.4 Hz), 8.26 (m, 1H, J = 7.3 Hz), 8.06 (d, 1H, J = 7.3 Hz), 8.01 (d, 1H, J = 8.1 Hz), 7.95 (d, 1H, J = 8.1 Hz), 7.79–7.87 (m, 2H), 7.37–7.57 (m, 8H), 7.31–7.37 (m, 3H), 6.00 (t, 1H, J = 9.5 Hz), 5.63 (s, 1H), 5.60 (m, 1H), 4.47 (m, 2H), 3.97 (t, 1H, J = 9.5 Hz), 3.85 (t, 1H, J = 10.3 Hz), 3.66–3.75 (m, 2H). ^{13}C NMR (100 MHz, $CDCl_3$) δ (ppm) 167.0, 166.2, 136.9, 134.1, 133.7, 133.6, 133.1, 131.4, 131.0, 130.9, 129.4, 129.0, 128.6, 128.4, 128.2, 128.0, 127.6, 126.3, 126.2, 126.1, 125.54,

125.46, 124.54, 124.48, 101.5, 79.2, 73.0, 71.8, 70.2, 68.7, 67.9. HRMS Calcd for $C_{35}H_{29}O_7$ $[M + H]^+$ 561.1913, found 561.1918.

Compound 16B. This product was isolated as a white crystalline solid in 37.2% yield. M.p. 93–95 °C. ^{1}H NMR ($CDCl_3$, 400MHz) δ 8.91 (d, 1H, J = 8.8 Hz), 8.21 (d, 1H, J = 7.3 Hz), 8.05 (d, 1H, J = 8.4 Hz), 7.89 (d, 1H, J = 8.1 Hz), 7.63 (m, 1H), 7.48-7.58 (m, 4H), 7.35–7.43 (m, 3H), 5.58 (s, 1H), 5.30 (m, 1H), 4.37 (m, 2H), 4.13 (dt, 1H, J = 2.6, 9.2 Hz), 3.76 (t, 1H, J = 10.3 Hz), 3.64 (t, 1H, J = 9.2 Hz), 3.42–3.54 (m, 2H), 2.83 (d, 1H, J = 2.6Hz). ^{13}C NMR ($CDCl_3$, 100 MHz) δ 166.9, 137.0, 133.8, 133.9, 131.3, 130.5, 129.3, 129.0, 128.4, 128.0, 126.4, 126.3, 125.7, 124.4, 102.0, 81.3, 72.9, 72.2, 71.1, 68.7, 67.4. HRMS Calcd for $C_{24}H_{23}O_6$ $[M + H]^+$ 407.1495, found 407.1491.

Synthesis of 8,10-diacetylene containing compounds 17A, 17B and 17C

Compound 17A. This product was isolated as a white crystalline solid in 6.0% yield. M.p. 45–47 °C. ^{1}H NMR (400 MHz, $CDCl_3$) δ 7.40–7.45 (m, 2H), 7.32–7.37 (m, 3H), 5.50 (s, 1H), 5.35 (t, 1H, J = 9.5 Hz), 5.05 (ddd-dt, 1H, J = 5.9, 9.5, 10.3 Hz), 4.34 (dd, 1H, J = 4.9, 10.5 Hz), 4.12 (dd, 1H, J = 5.9, 11.0 Hz), 3.72 (t, 1H, J = 10.3 Hz), 3.63 (t, 1H, J = 9.5 Hz), 3.47 (dt, 1H, J = 5.1, 9.7 Hz), 3.38 (t, 1H, J = 10.8 Hz), 2.14–2.37 (m, 12H), 1.58 (m, 4H), 1.41 (m, 8H), 1.38 (m, 4H), 1.26 (sb, 32H), 0.88 (t, 6H, J = 6.8 Hz). ^{13}C NMR (100 MHz, $CDCl_3$) δ (ppm) 172.71, 172.65, 136.8, 129.1, 128.2, 126.1, 101.4, 78.9, 77.7, 77.6, 77.2, 77.1, 72.1, 71.4, 69.4, 68.6, 67.5, 65.5, 65.4, 65.2, 65.1, 34.2, 33.9, 31.9, 29.6, 29.5, 29.3, 29.1, 28.9, 28.5, 28.3, 28.1, 28.0, 24.9, 24.6, 22.7, 19.2, 19.1, 14.1. HRMS Calcd for $C_{55}H_{80}O_7Na$ $[M + Na]^+$ 875.5802, found 875.5773.

Compound 17B. This product was isolated as a white crystalline solid in 32.6 % yield. M.p. 73–75 °C. ^{1}H NMR (400 MHz, $CDCl_3$) δ 7.45–7.52 (m, 2H), 7.33–7.41 (m, 3H), 5.55 (s, 1H), 4.95 (ddd, 1H, *J* = 5.9, 9.2, 10.6 Hz), 4.34 (dd, 1H, *J* = 4.5, 10.5 Hz), 4.12 (dd, 1H, *J* = 5.9, 11.0 Hz), 3.92 (t, 1H, *J* = 9.2 Hz), 3.71 (t, 1H, *J* = 10.3 Hz), 3.55 (t, 1H, *J* = 9.3 Hz), 3.40 (dt, 1H, *J* = 4.8, 9.7 Hz), 3.30 (t, 1H, *J* = 10.8 Hz), 2.29–2.43 (m, 2H), 2.20–2.29 (m, 4H), 1.64 (p, 2H *J* = 7.3 Hz), 1.51 (m, 4H), 1.32–1.45 (m, 2H), 1.26 (sb, 16H), 0.88 (t, 3H, *J* = 6.8 Hz). ^{13}C NMR (100 MHz, $CDCl_3$) δ (ppm) 173.2, 136.9, 129.3, 128.3, 126.2, 101.9, 81.2, 77.7, 77.1, 72.9, 71.5, 71.0, 68.7, 67.3, 65.4, 65.2, 34.0, 31.9, 29.5, 29.4, 29.3, 29.1, 28.8, 28.5, 28.4, 28.3, 28.1, 24.7, 24.5, 22.7, 19.2, 19.1, 14.1 HRMS Calcd for $C_{34}H_{48}O_6Na[M + Na]^+$ 573.3349, found 575.3344.

Compound 17C. This product was isolated as a white crystalline solid in 24.2% yield. M.p. 58–59 °C. ^{1}H NMR (400 MHz, $CDCl_3$) δ 7.42–7.47 (m, 2H), 7.32–7.39 (m, 3H), 5.51 (s, 1H), 5.02 (t, 1H, *J* = 9.2 Hz), 4.34 (dd, 1H, *J* = 4.9, 10.5 Hz), 4.10 (dd, 1H, *J* = 5.7, 11.5 Hz), 3.83 (ddd, 1H, *J* = 5.9, 9.2, 10.6 Hz), 3.70 (t, 1H, *J* = 10.3 Hz), 3.60 (t, 1H, *J* = 9.5 Hz), 3.44 (td, 1H, *J* = 4.8, 9.7 Hz), 3.39 (t, 1H, *J* = 10.8 Hz), 2.89 (sbr, 1H), 2.40 (t, 2H, *J* = 7.3 Hz), 2.24 (t, 2H, *J* = 7.0 Hz), 2.18 (t, 2H, *J* = 6.8 Hz), 1.64 (p, 2H, *J* = 7.3 Hz), 1.51 (p, 2H, *J* = 7.3 Hz), 1.19–1.45 (m, 20H), 0.88 (t, 3H, *J* = 6.8 Hz). ^{13}C NMR (100 MHz, $CDCl_3$) δ (ppm) 175.4, 137.0, 129.1, 128.2, 126.1, 101.5, 78.4, 77.7, 77.2, 77.0, 71.4, 70.6, 70.0, 68.8, 65.4, 65.2, 34.2, 31.8, 29.5, 29.4, 29.3, 29.0 28.8, 28.3, 27.9, 24.8, 22.6, 19.2, 19.0, 14.1. HRMS Calcd for $C_{34}H_{48}O_6Na$ $[M + Na]^+$ 573.3349, found 575.3336.

Synthesis of 10,12-diacetylene containing compounds 18A, 18B, and 18C

Compound 18A. This product was isolated as a white crystalline solid in 11.3% yield. M.p. 55–57 °C. ^{1}H NMR (400 MHz, $CDCl_3$) δ 7.40–7.45 (m, 2H), 7.32–7.37 (m, 3H), 5.50 (s, 1H), 5.35 (t, 1H, *J* = 9.5 Hz), 5.04 (ddd-dt, 1H, *J* = 5.9, 9.9, 10.3 Hz), 4.33 (dd, 1H, *J* = 4.9, 10.3 Hz), 4.12 (dd, 1H, *J* = 5.9, 11.0 Hz), 3.72 (t, 1H, *J* = 10.3 Hz), 3.63 (dd~t, 1H, *J* = 9.2, 9.9 Hz), 3.47 (dt, 1H, *J* = 5.1, 9.5 Hz), 3.38 (t, 1H, *J* = 10.8 Hz), 2.17–2.35 (m, 12H), 1.41–1.61 (m, 12H), 1.14–1.40 (m, 44H), 0.88 (t, 6H, *J* = 7.0 Hz). ^{13}C NMR (100 MHz, $CDCl_3$) δ (ppm) 172.81, 172.76, 136.9, 129.0, 128.2, 126.0, 101.4, 78.9, 77.6, 77.4, 72.1, 71.5, 69.4, 68.6, 67.5, 65.3, 65.2, 34.2, 34.0, 31.8, 29.5, 29.4, 29.3, 29.0, 28.9, 28.8, 28.7, 28.3, 28.2, 25.0, 24.7, 22.6, 19.1, 14.1. HRMS Calcd for $C_{59}H_{88}O_7Na$ $[M + Na]^+$ 931.6428, found 931.6392.

Compound 18B. This product was isolated as a white crystalline solid in 46.4% yield. M.p. 88–90 °C. ^{1}H NMR (400 MHz, $CDCl_3$) δ 7.45–7.51 (m, 2H), 7.33–7.41 (m, 3H), 5.55 (s, 1H), 4.95 (ddd, 1H, *J* = 5.9, 9.2, 10.6 Hz), 4.33 (dd, 1H, *J* = 5.1, 10.6 Hz), 4.12 (dd, 1H, *J* = 5.9, 11.0 Hz), 3.91 (t, 1H, *J* = 9.2 Hz), 3.71 (t, 1H, *J* = 10.3 Hz), 3.54 (t, 1H, *J* = 9.2 Hz), 3.40 (dt, 1H, *J* = 4.9, 9.7 Hz), 3.30 (t, 1H, *J* = 10.8 Hz), 2.29–2.42 (m, 2H), 2.24 (t, 4H, *J* = 6.8 Hz), 1.63 (m, 2H), 1.52 (p, 4H, *J* = 7.3 Hz), 1.17–1.43 (m, 22H), 0.88 (t, 3H, *J* = 6.8 Hz). ^{13}C NMR (100 MHz, $CDCl_3$) δ (ppm) 172.3, 136.9, 129.3, 128.3, 126.2, 101.9, 81.2, 77.6, 77.4, 72.8, 71.5, 71.0, 68.6, 67.3, 65.3, 65.2, 34.1, 31.9, 29.54, 29.46, 29.3, 29.1, 28.9, 28.85, 28.7, 28.32, 28.26, 24.8, 22.7, 19.2, 14.1. HRMS Calcd for $C_{36}H_{52}O_6Na$ $[M + Na]^+$ 603.3662, found 603.3657.

Compound 18C. This product was isolated as a white crystalline solid in 26.9 % yield. M.p. 41–43 °C. ^{1}H NMR (400 MHz, $CDCl_3$) δ 7.42–7.47 (m, 2H), 7.32–7.38 (m, 3H), 5.50 (s, 1H), 5.03

(t, 1H, *J* = 9.2 Hz), 4.33 (dd, 1H, *J* = 4.9, 10.5 Hz), 4.09 (dd, 1H, *J* = 5.7, 11.4 Hz), 3.82 (m, 1H), 3.70 (t, 1H, *J* = 10.3 Hz), 3.59 (t, 1H, *J* = 9.5 Hz), 3.44 (td, 1H, *J* = 4.8, 9.5 Hz), 3.38 (t, 1H, *J* = 10.8 Hz), 2.40 (t, 2H, *J* = 7.5 Hz), 2.16–2.30 (m, 4H), 1.63 (m, 2H), 1.42–1.56 (m, 4H), 1.17–1.43 (m, 22H), 0.88 (t, 3H, *J* = 7.0 Hz). ^{13}C NMR (100 MHz, $CDCl_3$) δ (ppm) 175.5, 137.0, 129.1, 128.2, 126.1, 101.4, 78.4, 77.6, 77.4, 76.9, 71.3, 70.6, 70.0, 68.7, 65.3, 65.2, 34.3, 31.8, 29.5, 29.4, 29.3, 29.0 (2), 28.8, 28.75, 28.7, 28.3, 28.2, 24.9, 22.6, 19.2, 19.1, 14.1. HRMS Calcd for $C_{36}H_{52}O_6Na$ $[M + Na]^+$ 603.3662, found 603.3656.
