# Supplementary material for: Synthesis and self-assembly of 1-deoxyglucose derivatives as low molecular weight organogelators
Source: Beilstein J Org Chem. 2011 Feb 21;7:234–42. doi: 10.3762/bjoc.7.31 (PMC3062983; doi:10.3762/bjoc.7.31)
Supplement: File 2 — 1H and 13C NMR spectra of compounds 9A–18C. [file Beilstein_J_Org_Chem-07-234-s002.pdf]

Supporting Information

for

Synthesis and self-assembly of 1-deoxyglucose derivatives as low molecular weight organogelators

Guijun Wang*[1], Hao Yang[1], Sherwin Cheuk[1] and Sherman Coleman[2]

Address: [1]Department of Chemistry, University of New Orleans, New Orleans, LA 70148, Phone: 504 280-1258, Fax: 504 280-6860 and [2]Dillard University, 2601 Gentilly Boulevard, New Orleans, Louisiana 70122

Email: Guijun Wang - gwang2@uno.edu

*Corresponding author

^{1}H and ^{13}C NMR spectra of compounds 9A–18C

Table of contents……………………………………………………….…………………......S1

^{1}H and ^{13}C NMR spectra of compound **9A**…..........................…………………….S3

^{1}H and ^{13}C NMR spectra of compound **9B**……………………………………..….S4

^{1}H and ^{13}C NMR spectra of compound **9C**……………………………………........S5

^{1}H and ^{13}C NMR spectra of compound **10A**………………………………………….S6

^{1}H and ^{13}C NMR spectra of compound **10B**………………………………………….S7

^{1}H and ^{13}C NMR spectra of compound **10C**……………………………………………S8
^{1}H and ^{13}C NMR spectra of compound **11A**……………………………………………S9
^{1}H and ^{13}C NMR spectra of compound **11B**……………………………………………S10
^{1}H and ^{13}C NMR spectra of compound **11C**……………………………………………S11
^{1}H and ^{13}C NMR spectra of compound **12A**……………………………………………S12
^{1}H and ^{13}C NMR spectra of compound **12B**……………………………………………S13
^{1}H and ^{13}C NMR spectra of compound **12C**……………………………………………S14
^{1}H and ^{13}C NMR spectra of compound **13A**……………………………………………S15
^{1}H and ^{13}C NMR spectra of compound **13B**……………………………………………S16
^{1}H and ^{13}C NMR spectra of compound **13C**……………………………………………S17
^{1}H and ^{13}C NMR spectra of compound **14A**……………………………………………S18
^{1}H and ^{13}C NMR spectra of compound **14B**……………………………………………S19
^{1}H and ^{13}C NMR spectra of compound **14C**……………………………………………S20
^{1}H and ^{13}C NMR spectra of compound **15A**……………………………………………S21
^{1}H and ^{13}C NMR spectra of compound **15B**……………………………………………S22
^{1}H and ^{13}C NMR spectra of compound **15C**……………………………………………S23
^{1}H and ^{13}C NMR spectra of compound **16A**……………………………………………S24
^{1}H and ^{13}C NMR spectra of compound **16B**……………………………………………S25
^{1}H and ^{13}C NMR spectra of compound **17A**……………………………………………S26
^{1}H and ^{13}C NMR spectra of compound **17B**……………………………………………S27
^{1}H and ^{13}C NMR spectra of compound **17C**……………………………………………S28
^{1}H and ^{13}C NMR spectra of compound **18A**……………………………………………S29
^{1}H and ^{13}C NMR spectra of compound **18B**……………………………………………S30
^{1}H and ^{13}C NMR spectra of compound **18C**……………………………………………S31

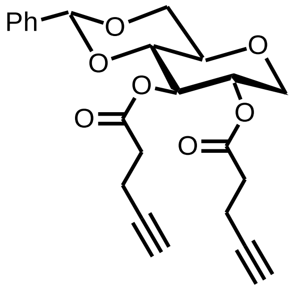

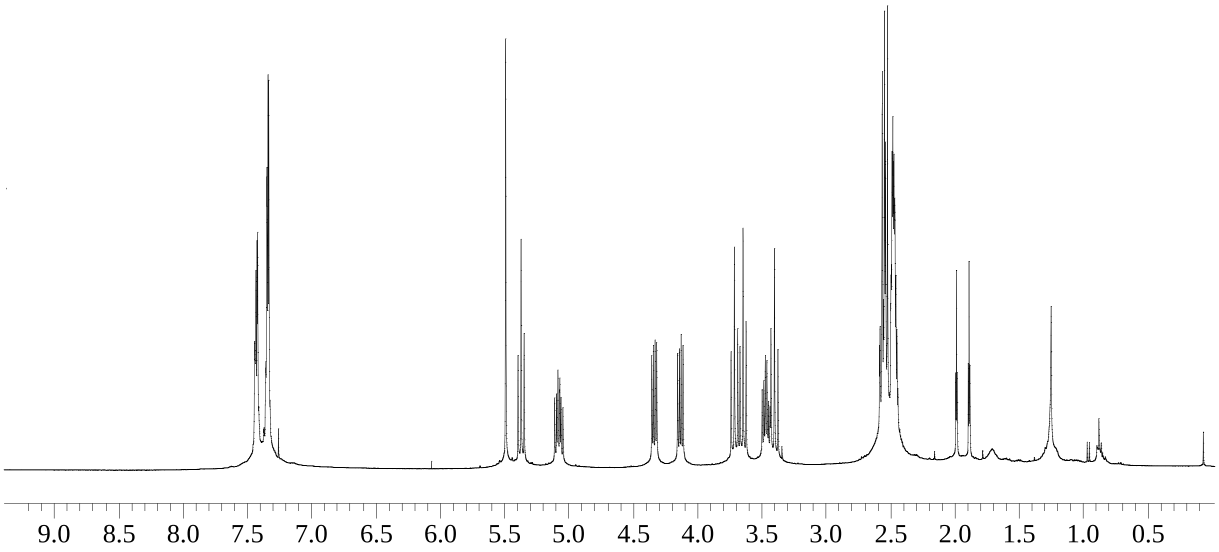

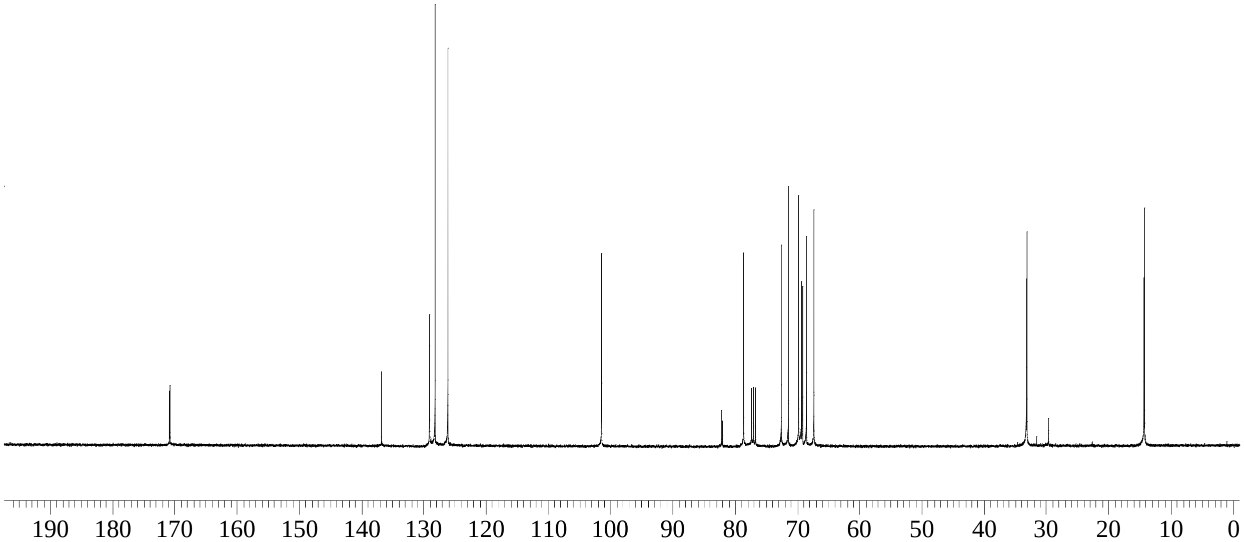

^{1}H NMR (400 MHz, $CDCl_3$) and ^{13}C NMR (100 MHz, $CDCl_3$) spectra of compound **9A.**

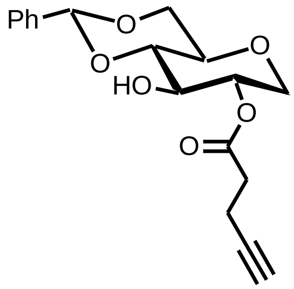

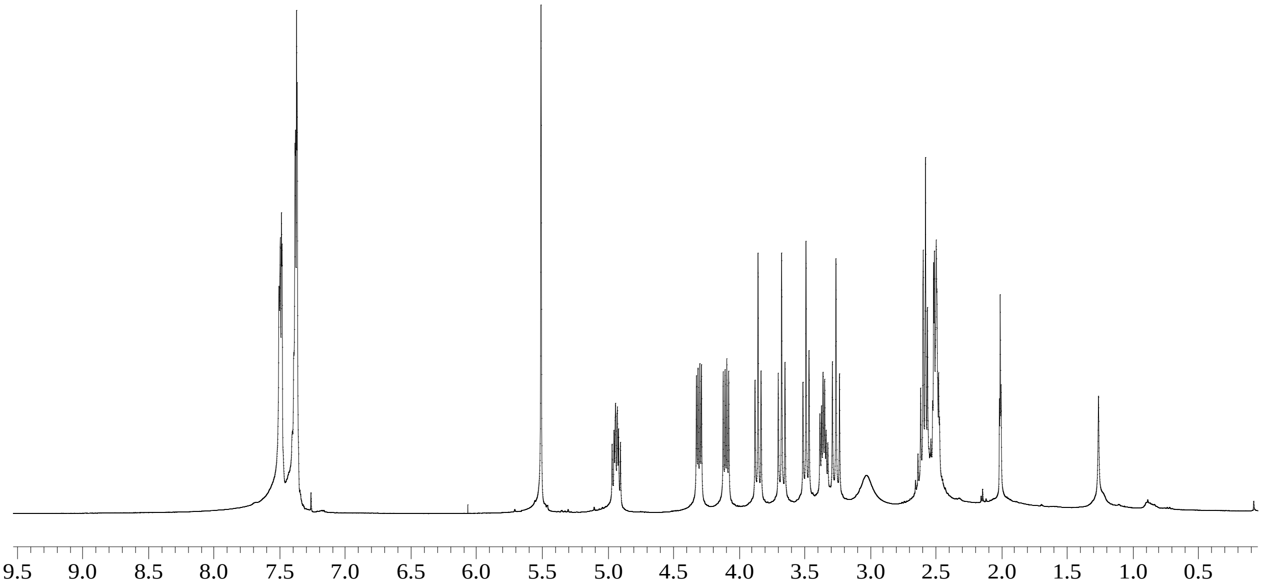

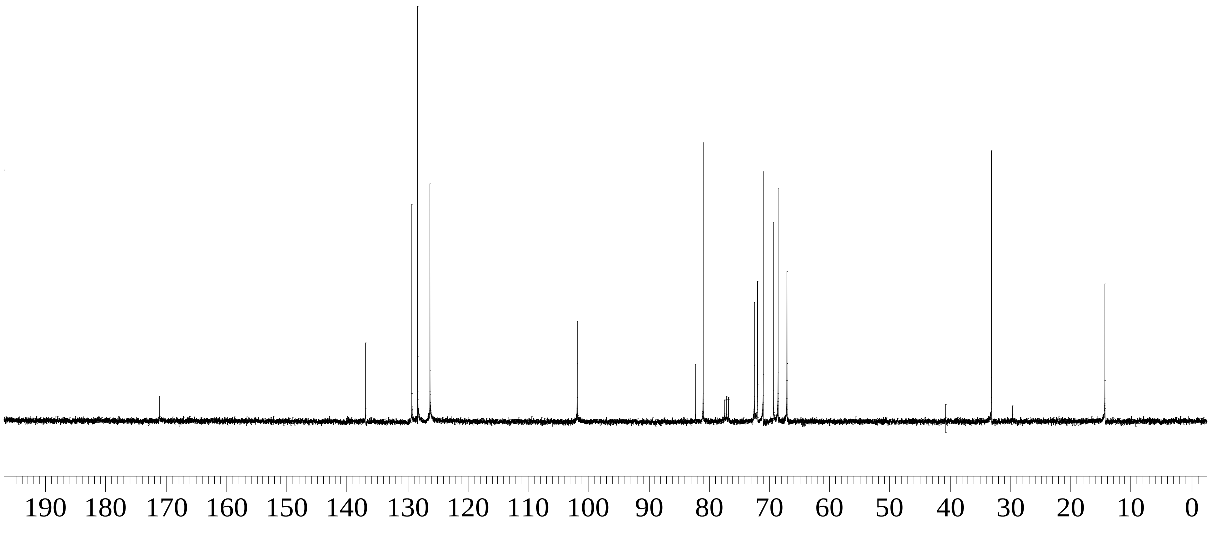

^{1}H NMR (400 MHz, $CDCl_3$) and ^{13}C NMR (100 MHz, $CDCl_3$) spectra of compound **9B.**

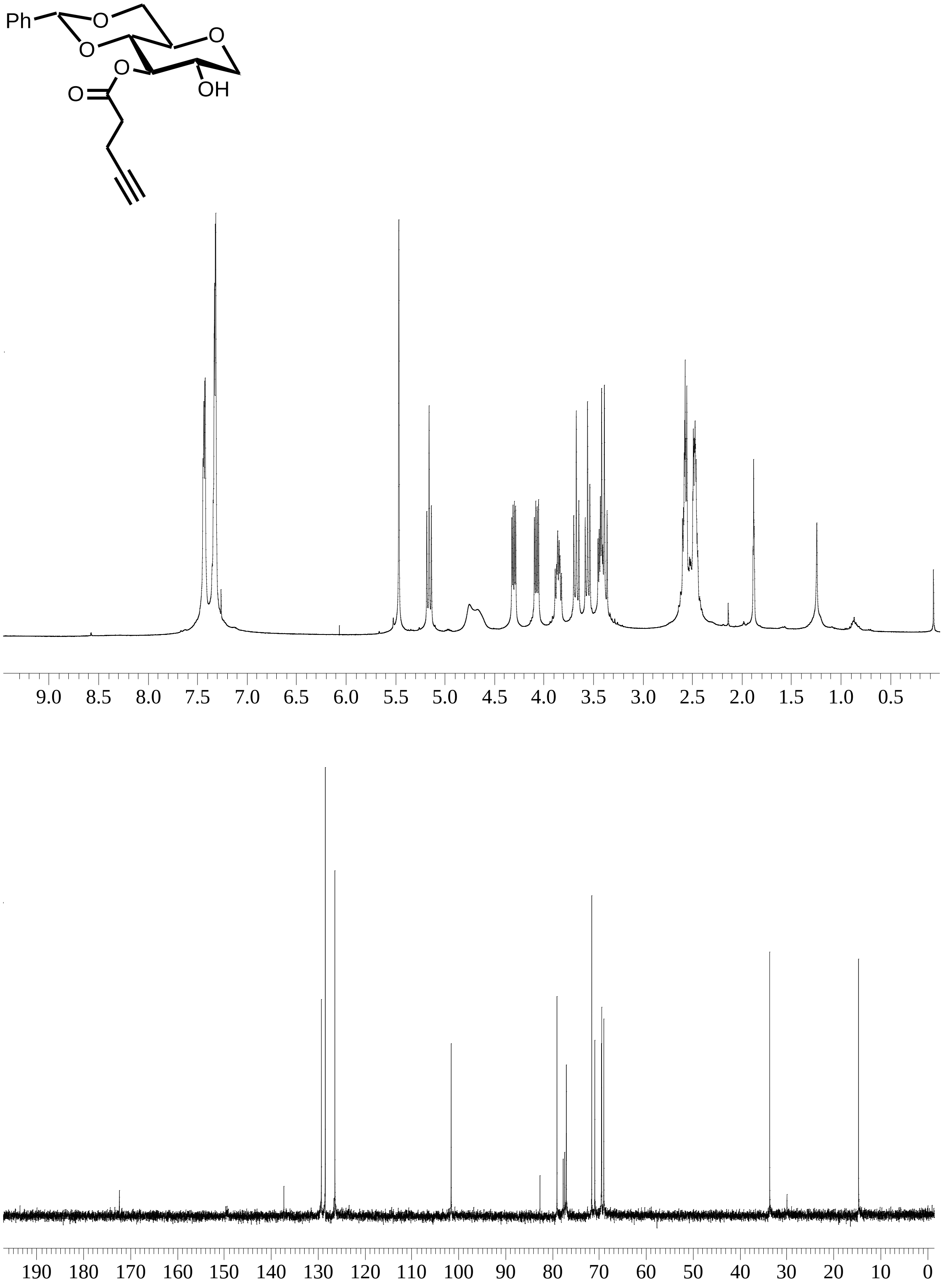

^{1}H NMR (400 MHz, $CDCl_3$) and ^{13}C NMR (100 MHz, $CDCl_3$) spectra of compound **9C.**

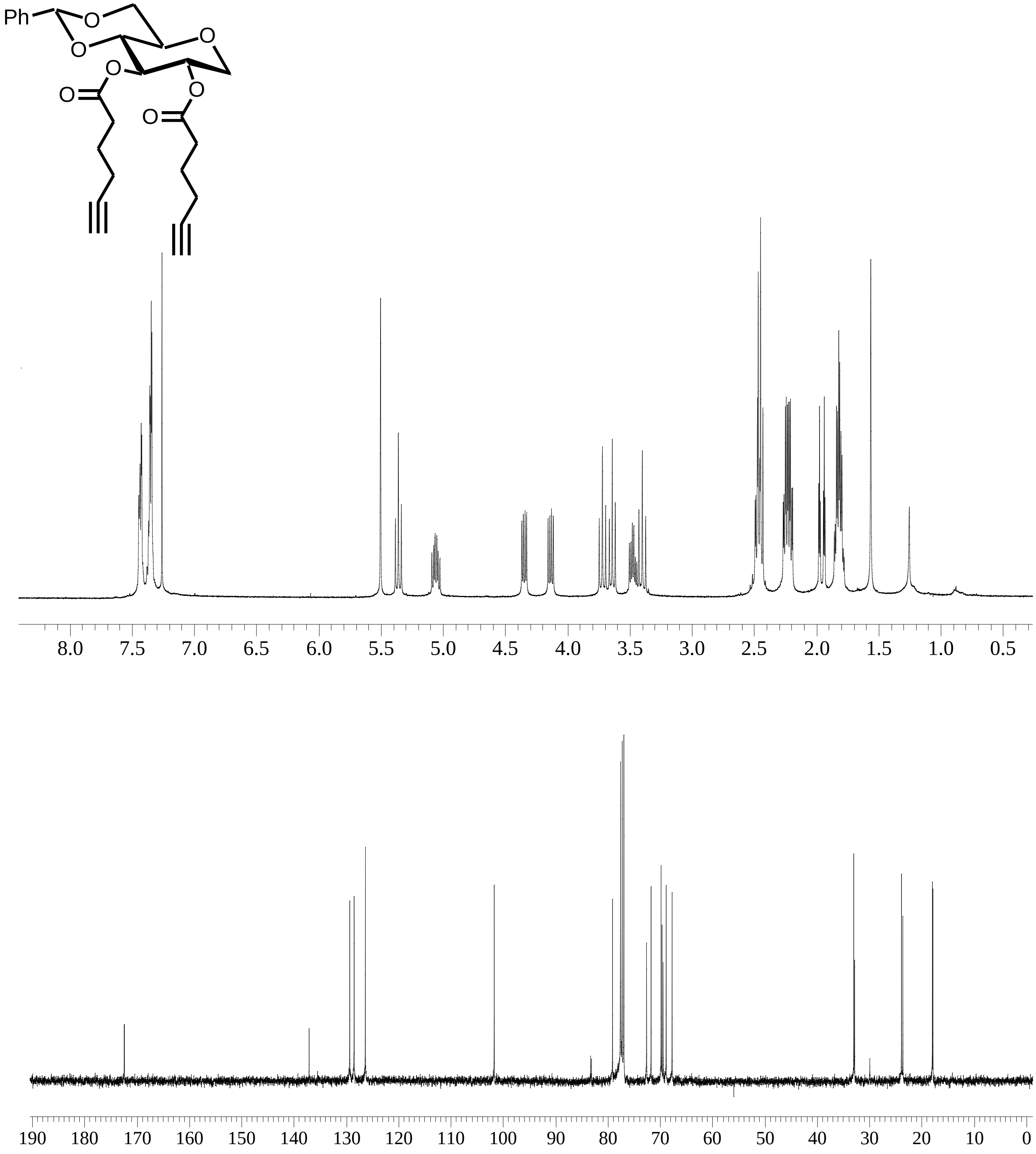

^{1}H NMR (400 MHz, $CDCl_3$) and ^{13}C NMR (100 MHz, $CDCl_3$) spectra of compound **10A.**

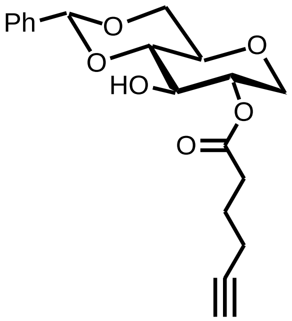

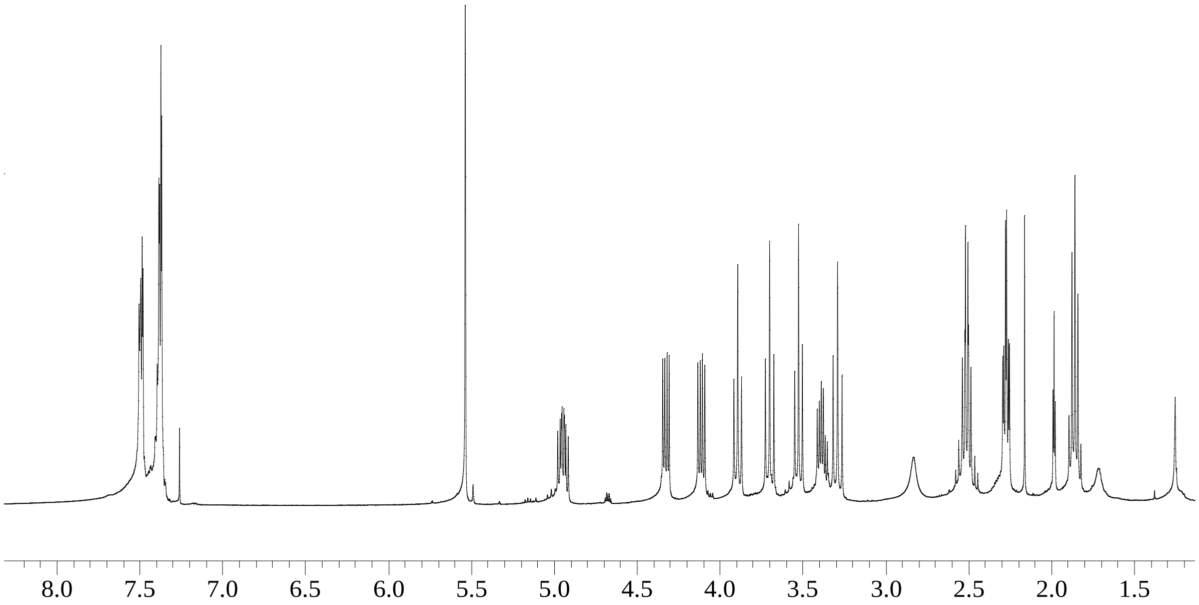

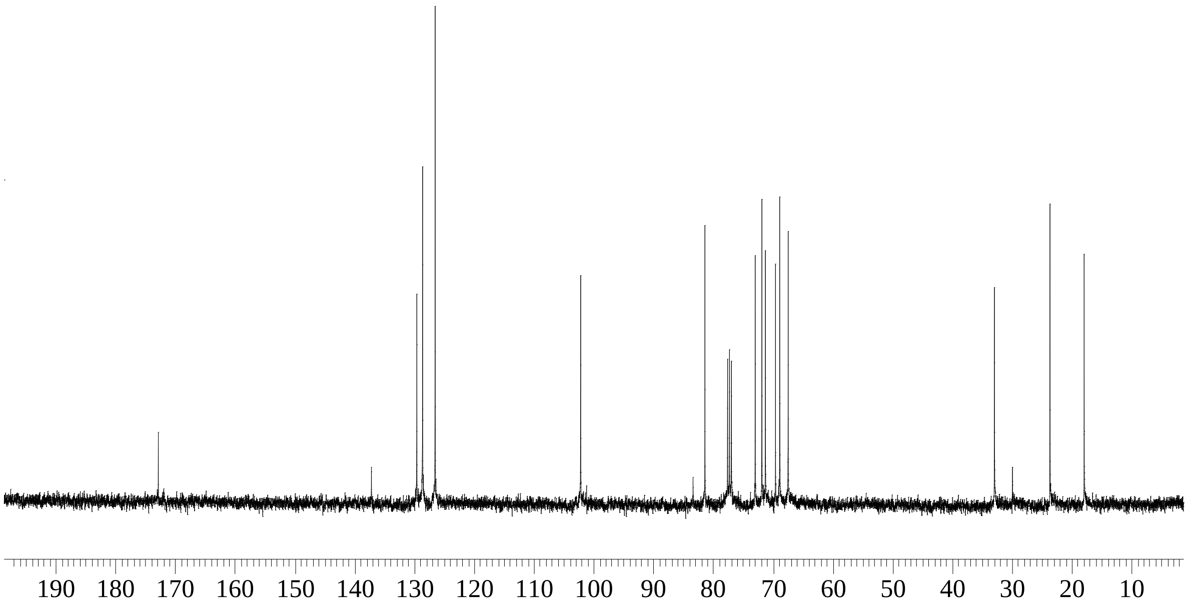

^{1}H NMR (400 MHz, $CDCl_3$) and ^{13}C NMR (100 MHz, $CDCl_3$) spectra of compound **10B.**

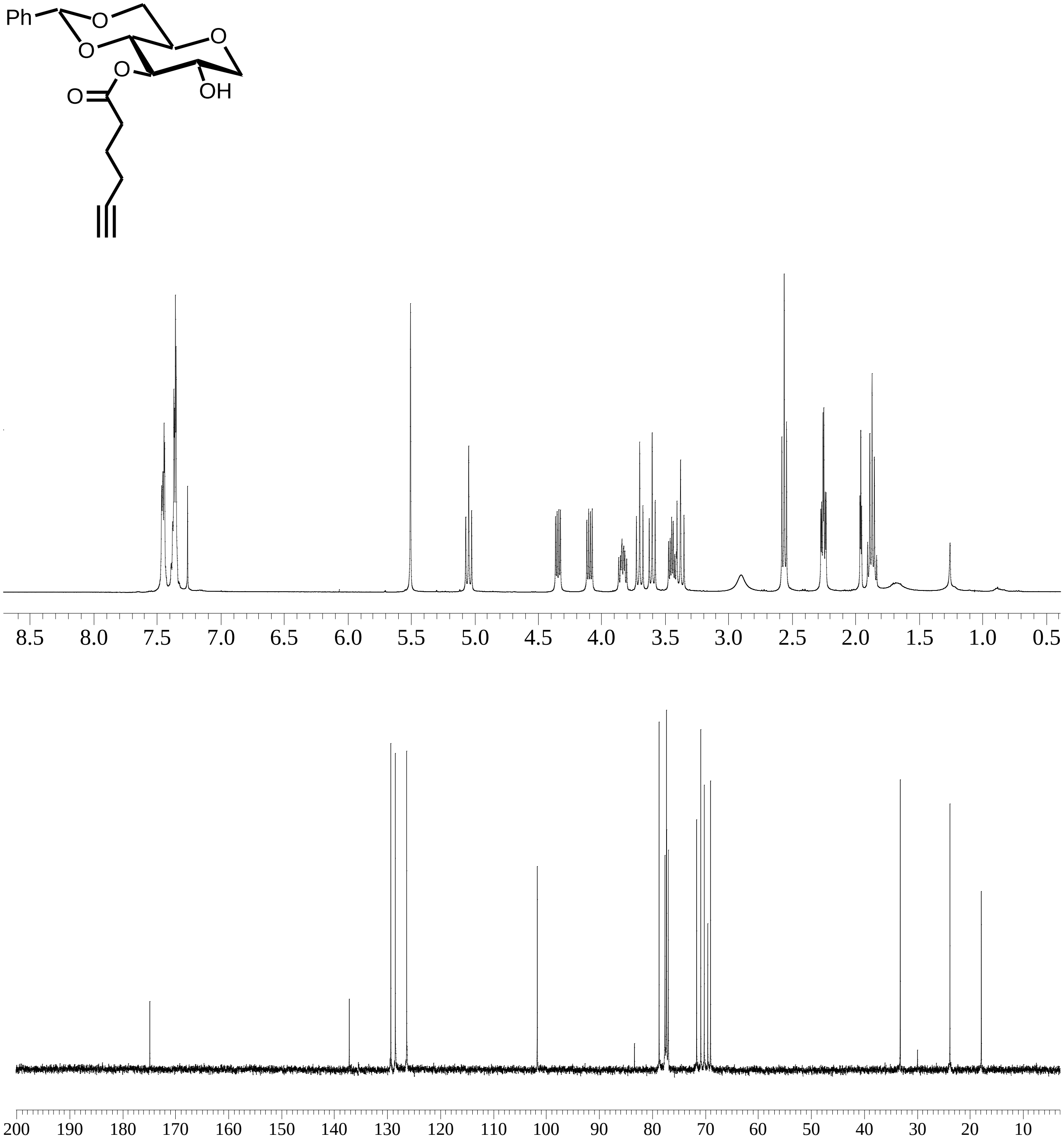

^{1}H NMR (400 MHz, $CDCl_3$) and ^{13}C NMR (100 MHz, $CDCl_3$) spectra of compound **10C.**

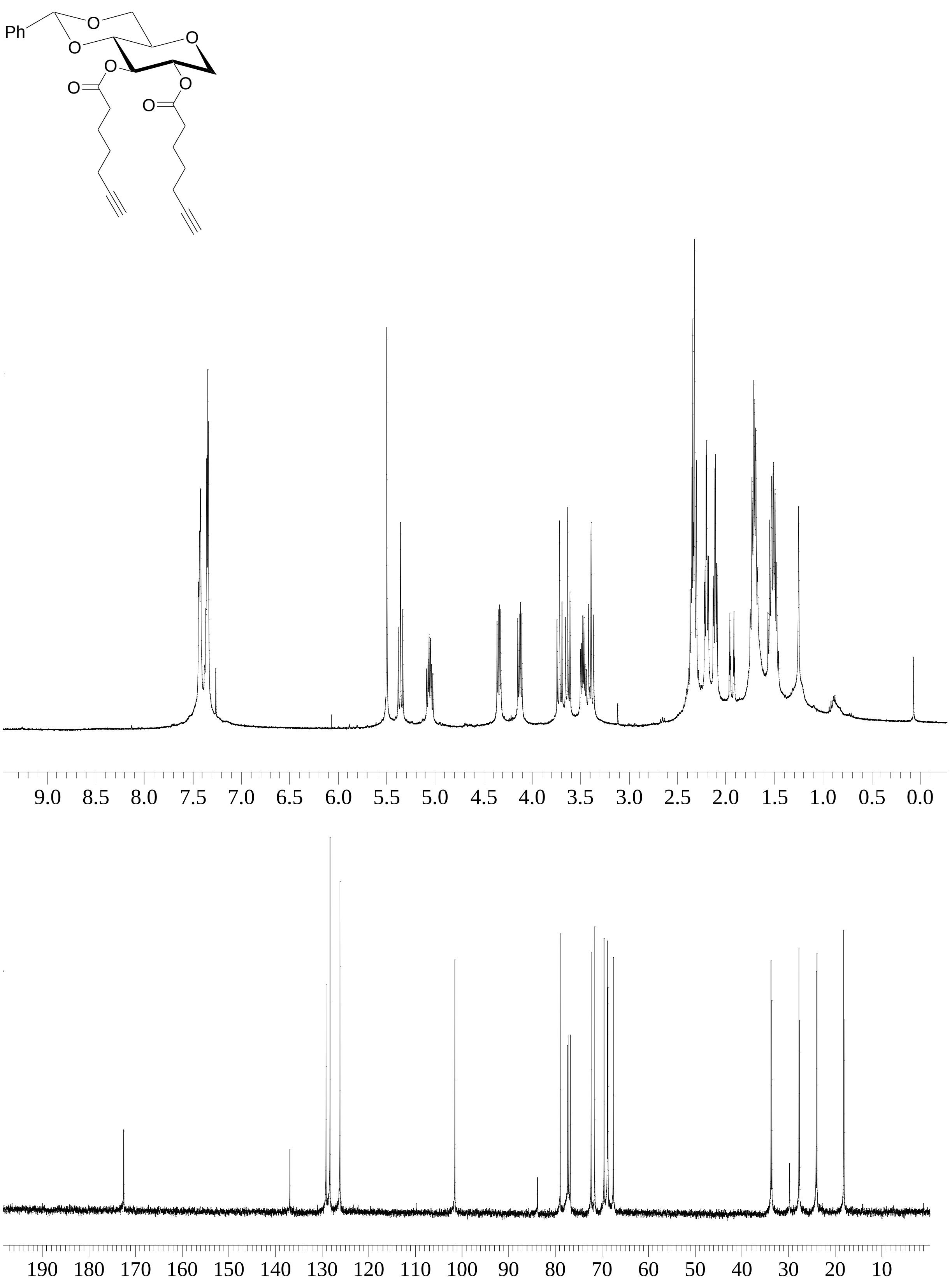

^{1}H (400 MHz, $CDCl_3$) and ^{13}C NMR (100 MHz, $CDCl_3$) spectra of compound **11A.**

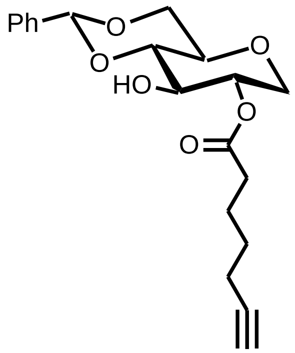

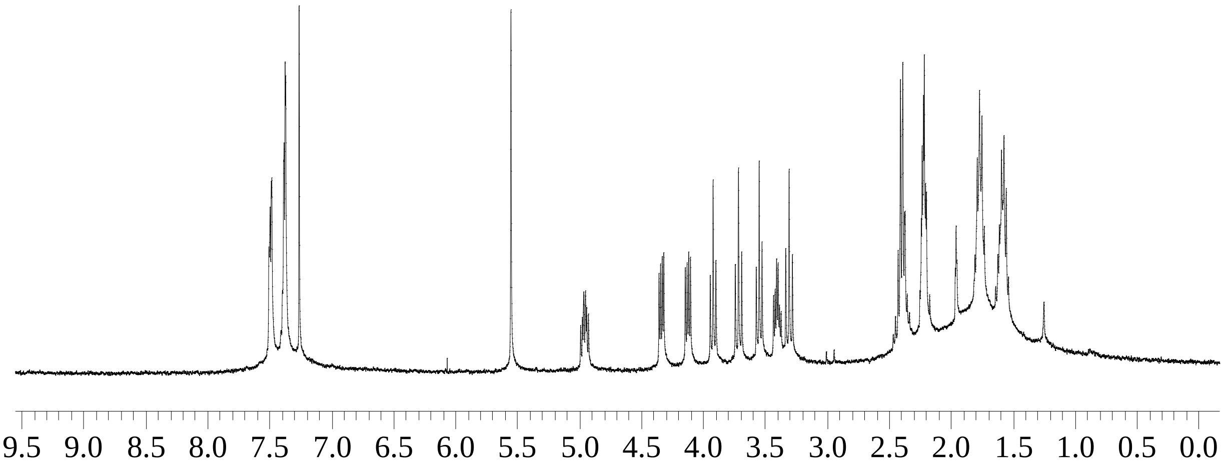

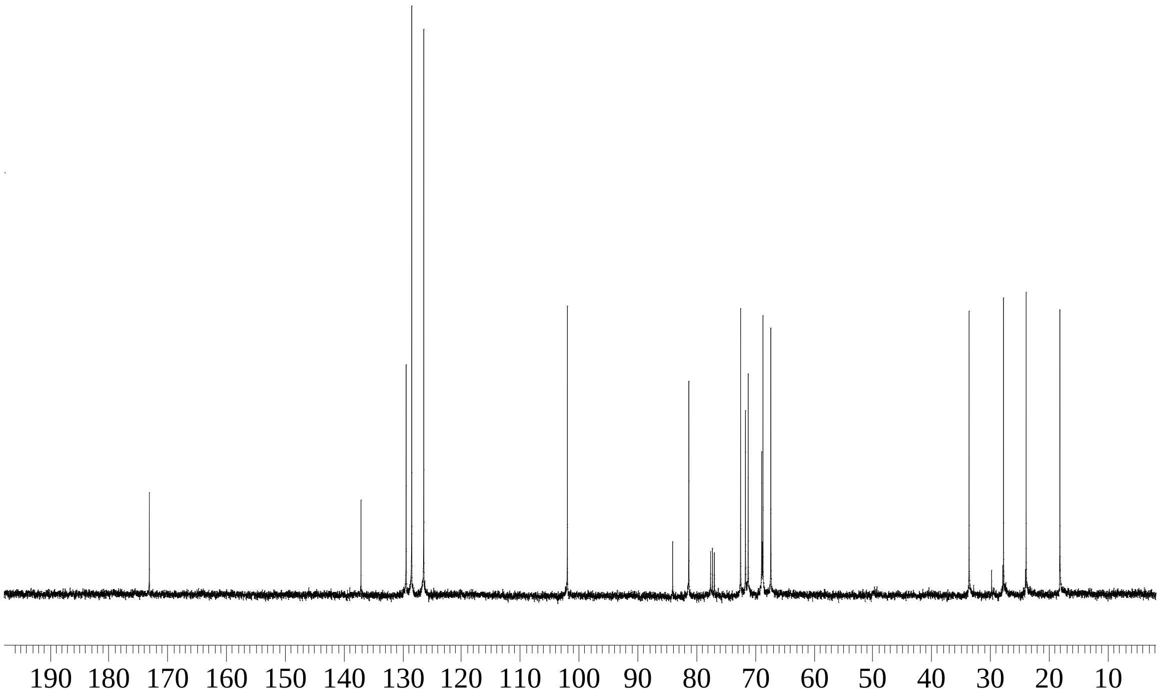

^{1}H NMR (400 MHz, $CDCl_3$) and ^{13}C NMR (100 MHz, $CDCl_3$) spectra of compound **11B.**

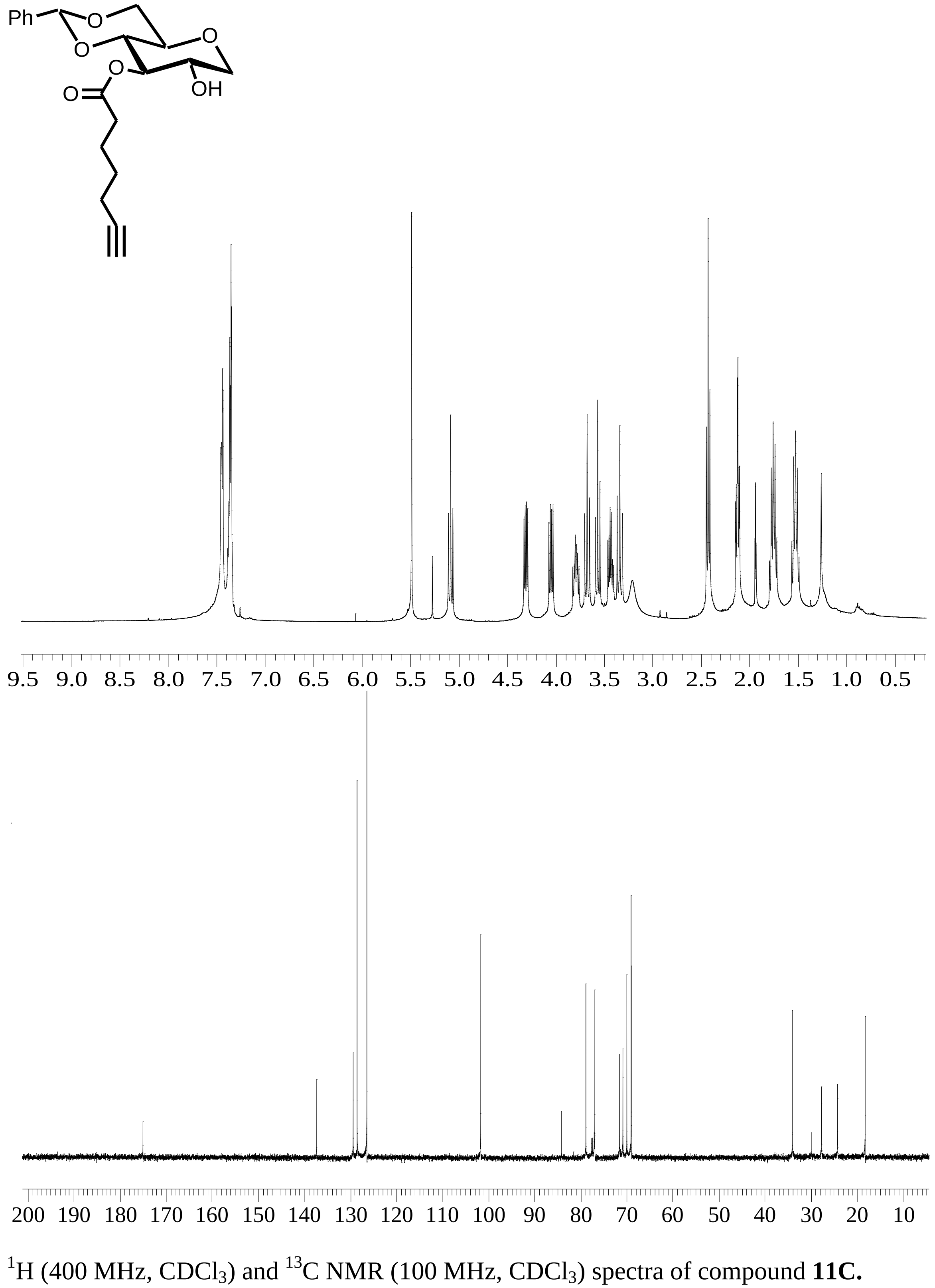

^{1}H (400 MHz, $CDCl_3$) and ^{13}C NMR (100 MHz, $CDCl_3$) spectra of compound **11C.**

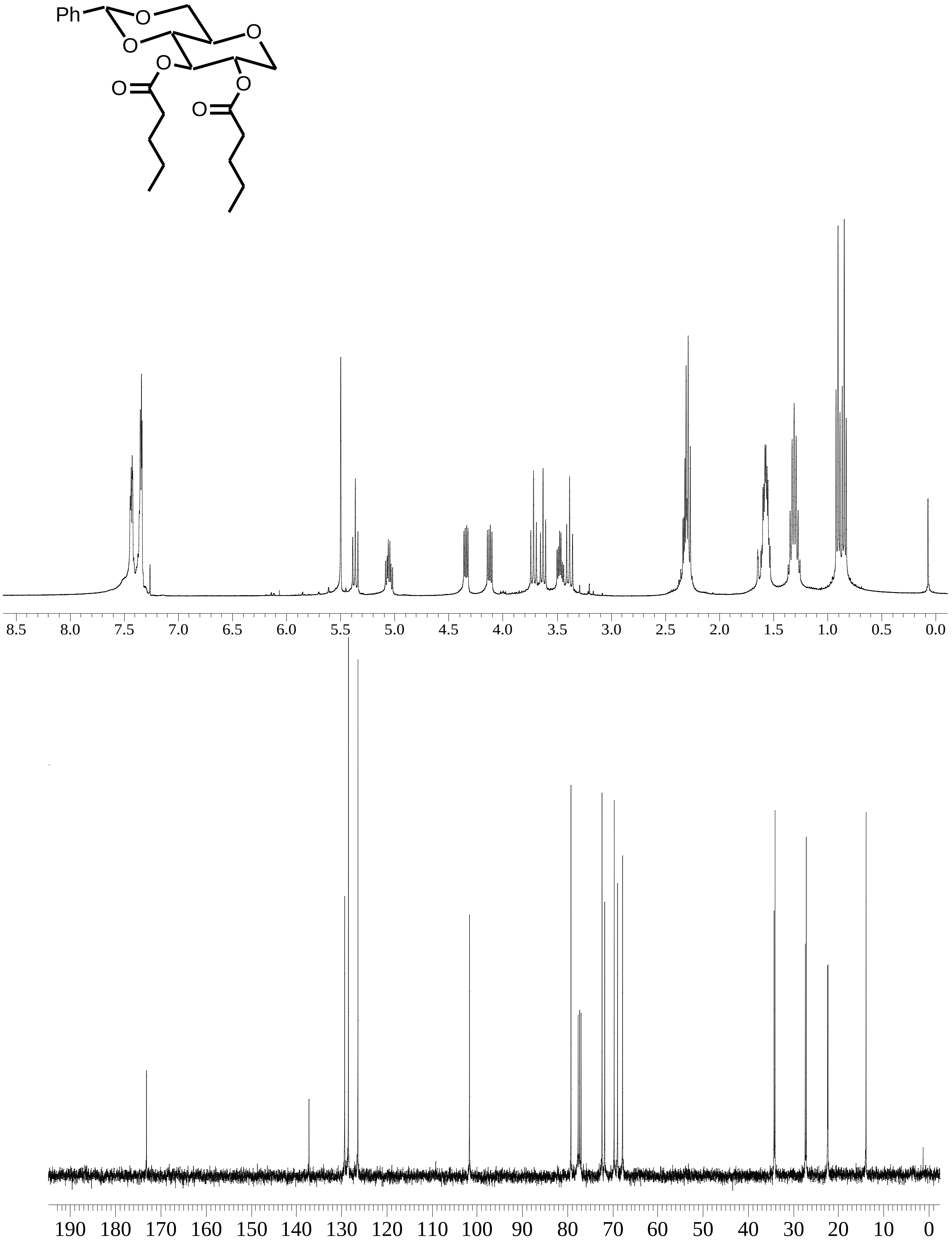

^{1}H (400 MHz, $CDCl_3$) and ^{13}C NMR (100 MHz, $CDCl_3$) spectra of compound **12A.**

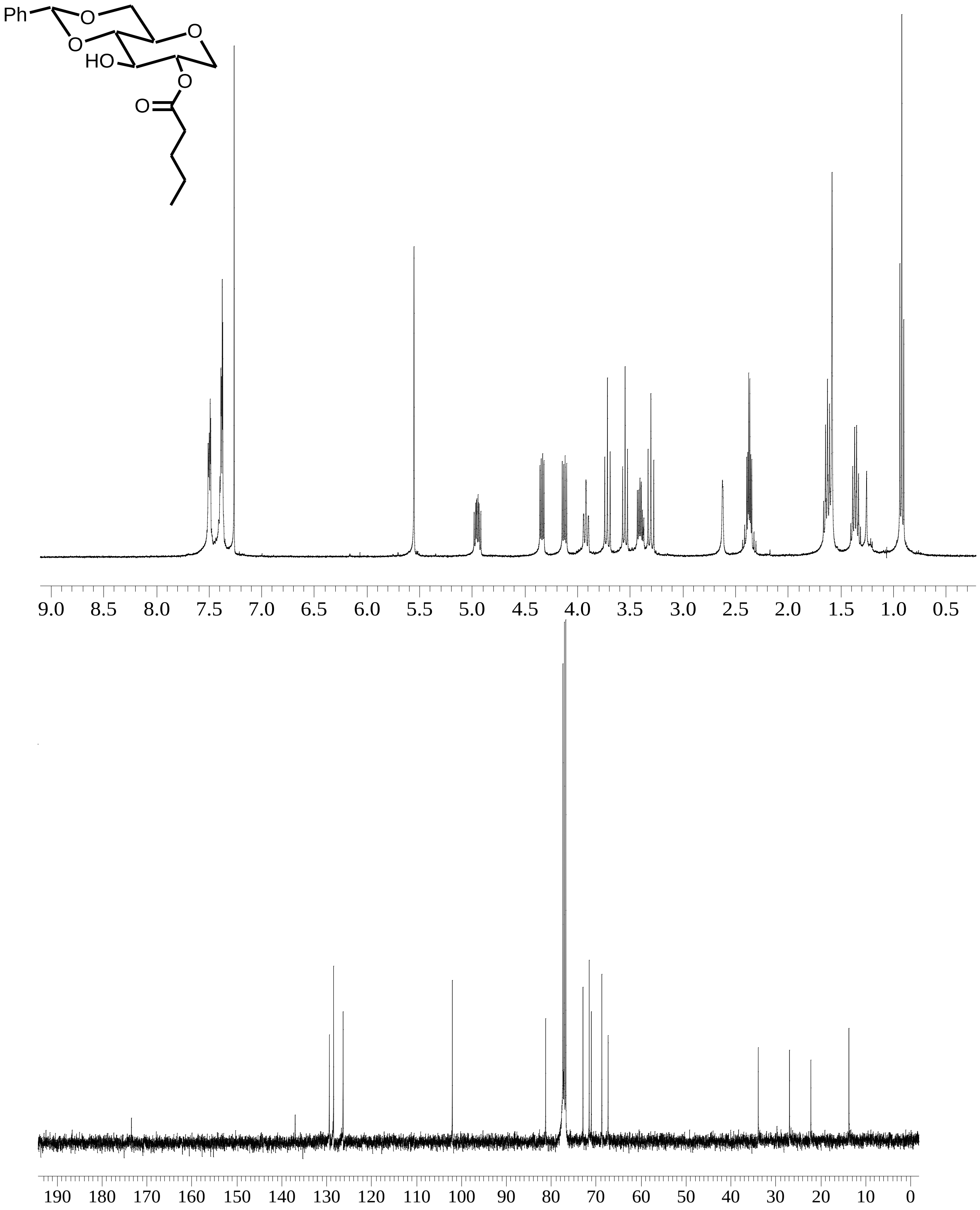

^{1}H (400 MHz, $CDCl_3$) and ^{13}C NMR (100 MHz, $CDCl_3$) spectra of compound **12B.**

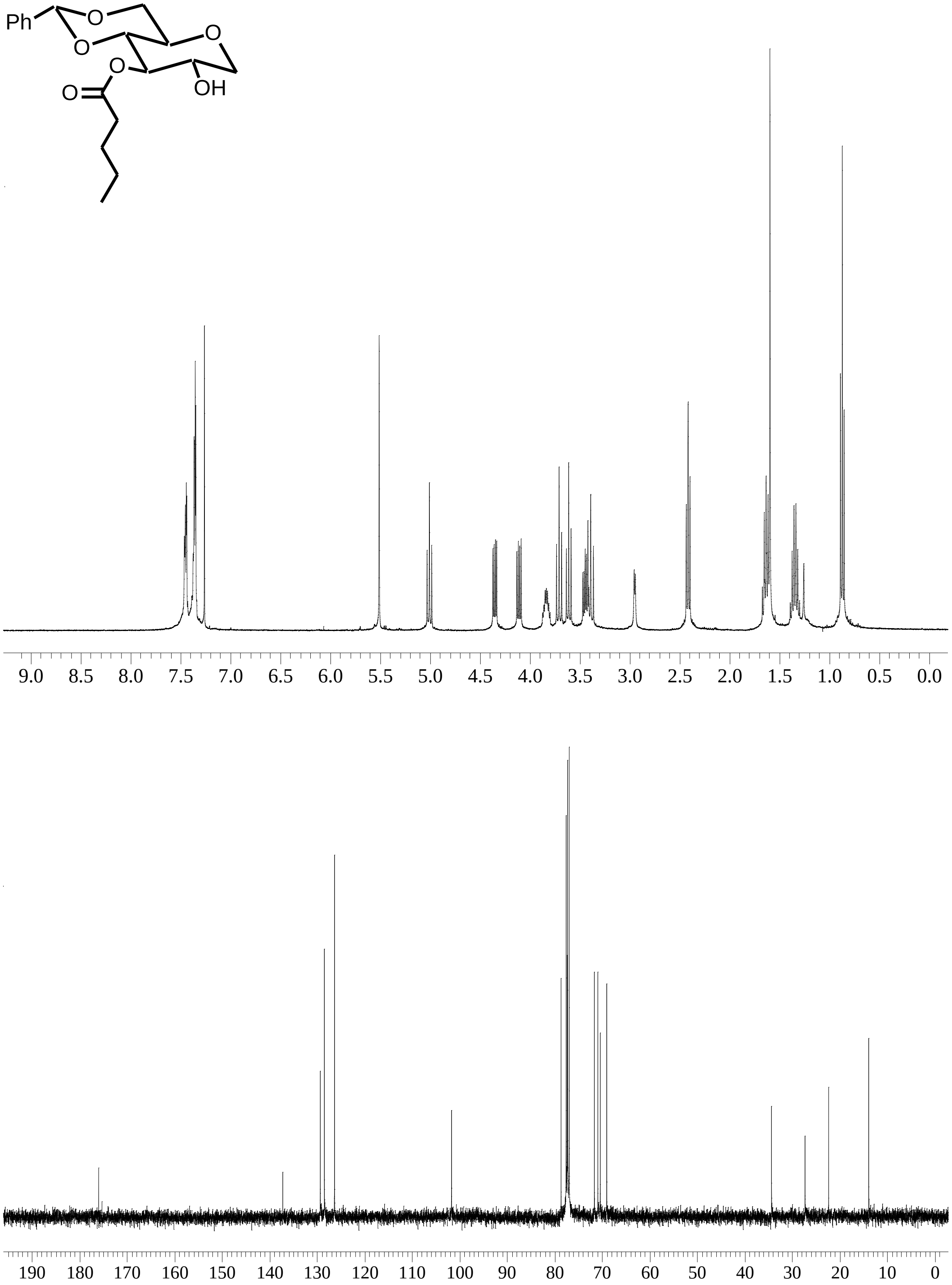

^{1}H (400 MHz, $CDCl_3$) and ^{13}C NMR (100 MHz, $CDCl_3$) spectra of compound **12C.**

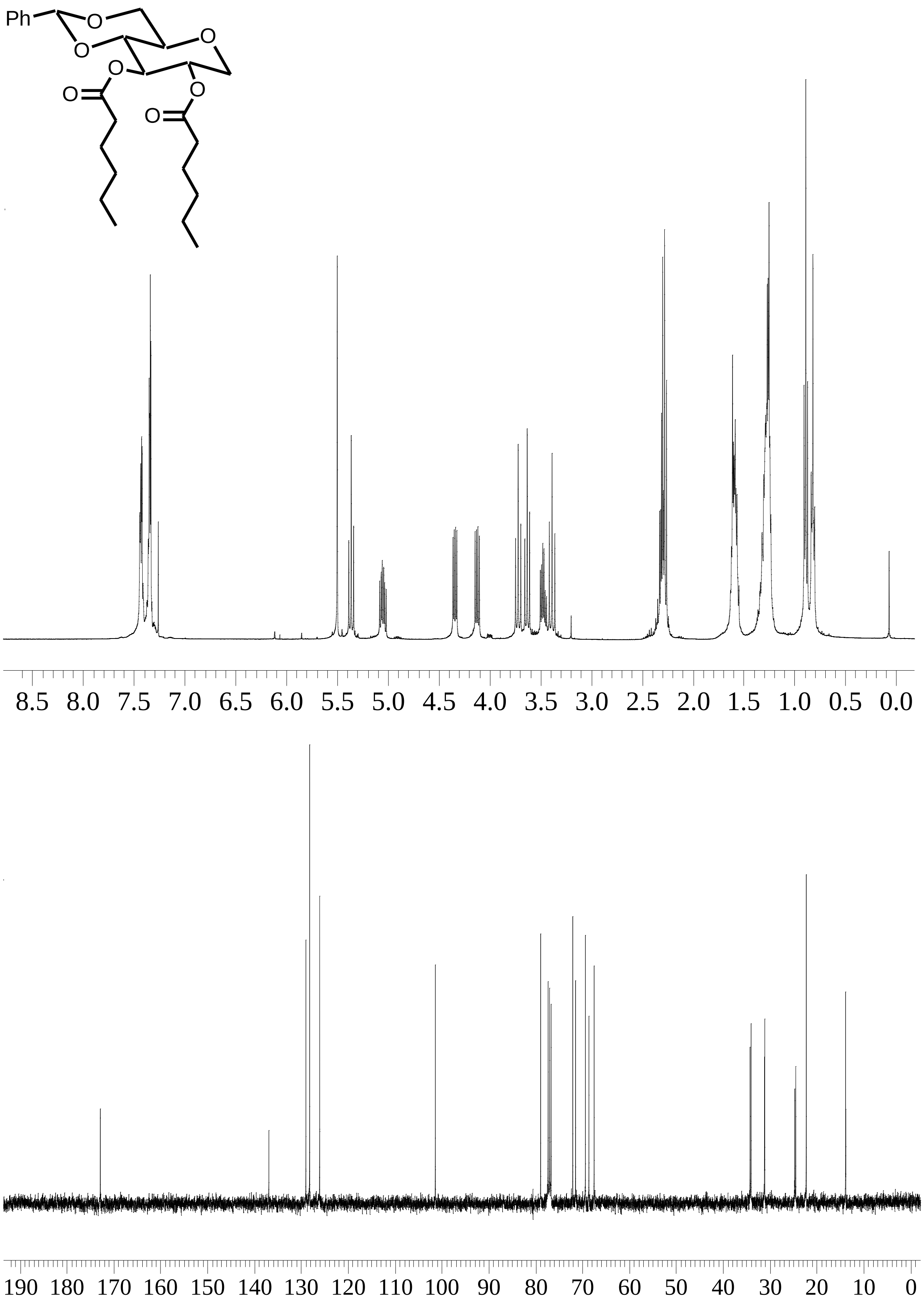

^{1}H (400 MHz, $CDCl_3$) and ^{13}C NMR (100 MHz, $CDCl_3$) spectra of compound **13A.**

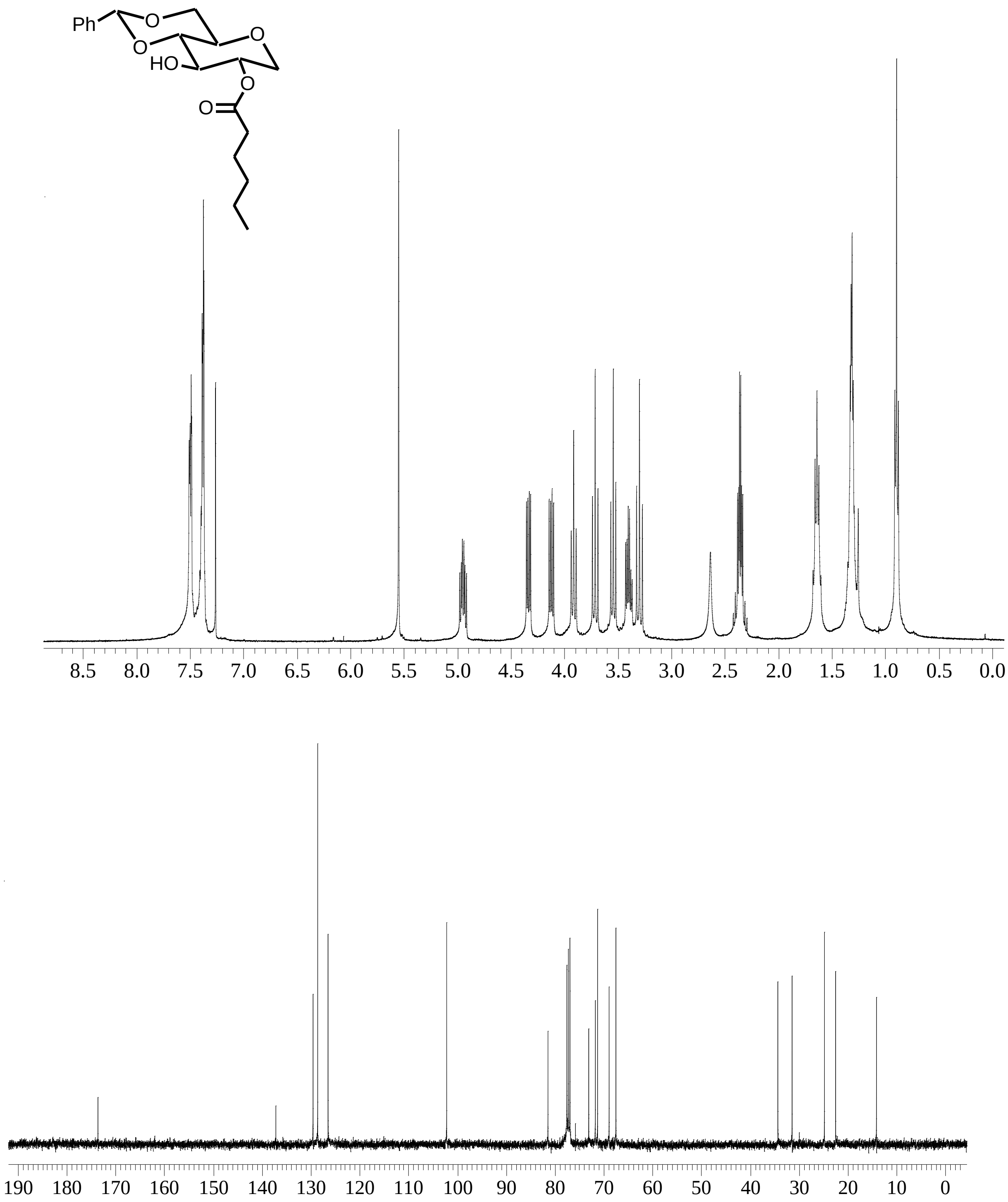

^{1}H NMR (400 MHz, $CDCl_3$) and ^{13}C NMR (100 MHz, $CDCl_3$) spectra of compound **13B.**

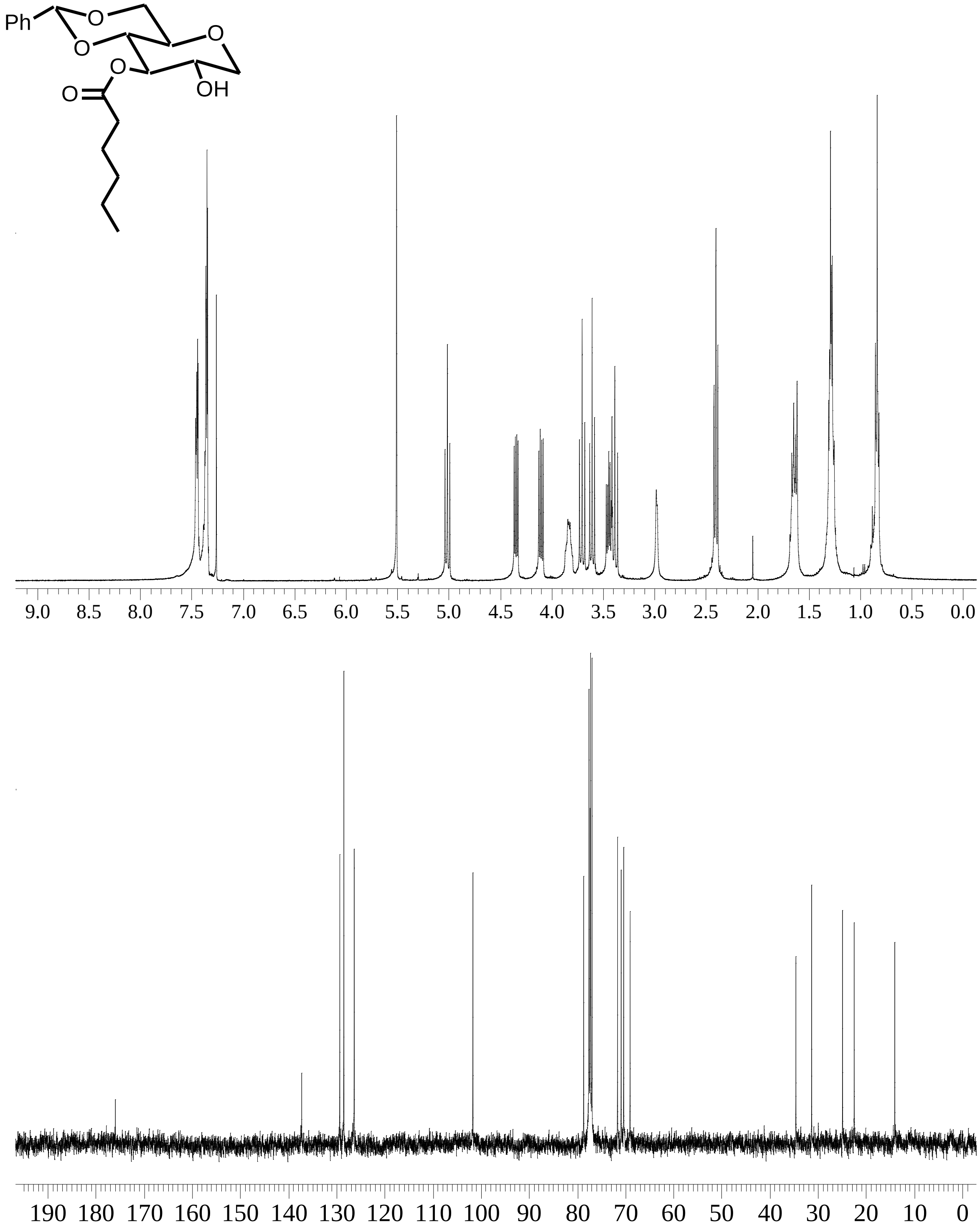

^{1}H (400 MHz, $CDCl_3$) and ^{13}C NMR (100 MHz, $CDCl_3$) spectra of compound **13C.**

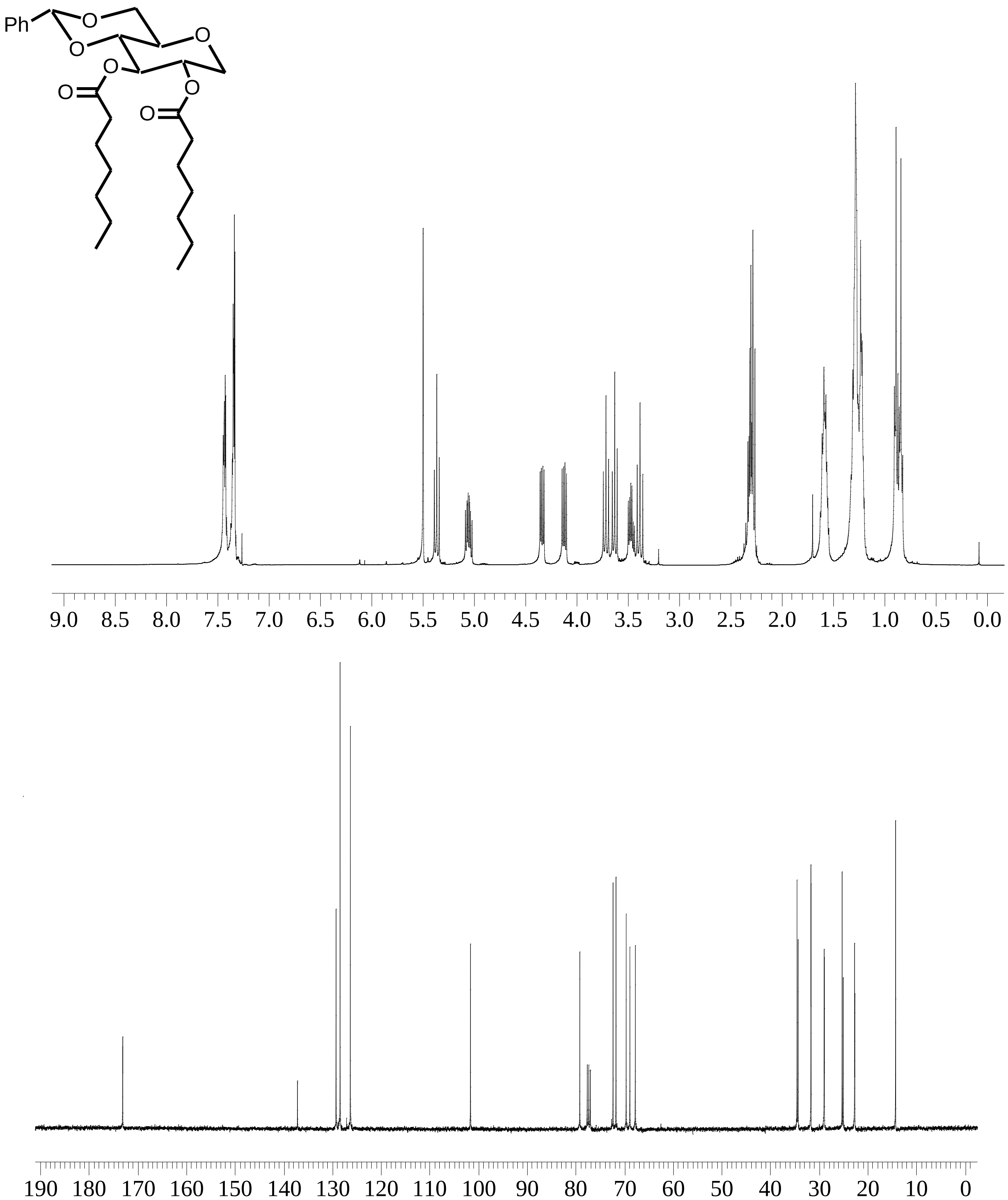

^{1}H (400 MHz, $CDCl_3$) and ^{13}C NMR (100 MHz, $CDCl_3$) spectra of compound **14A.**

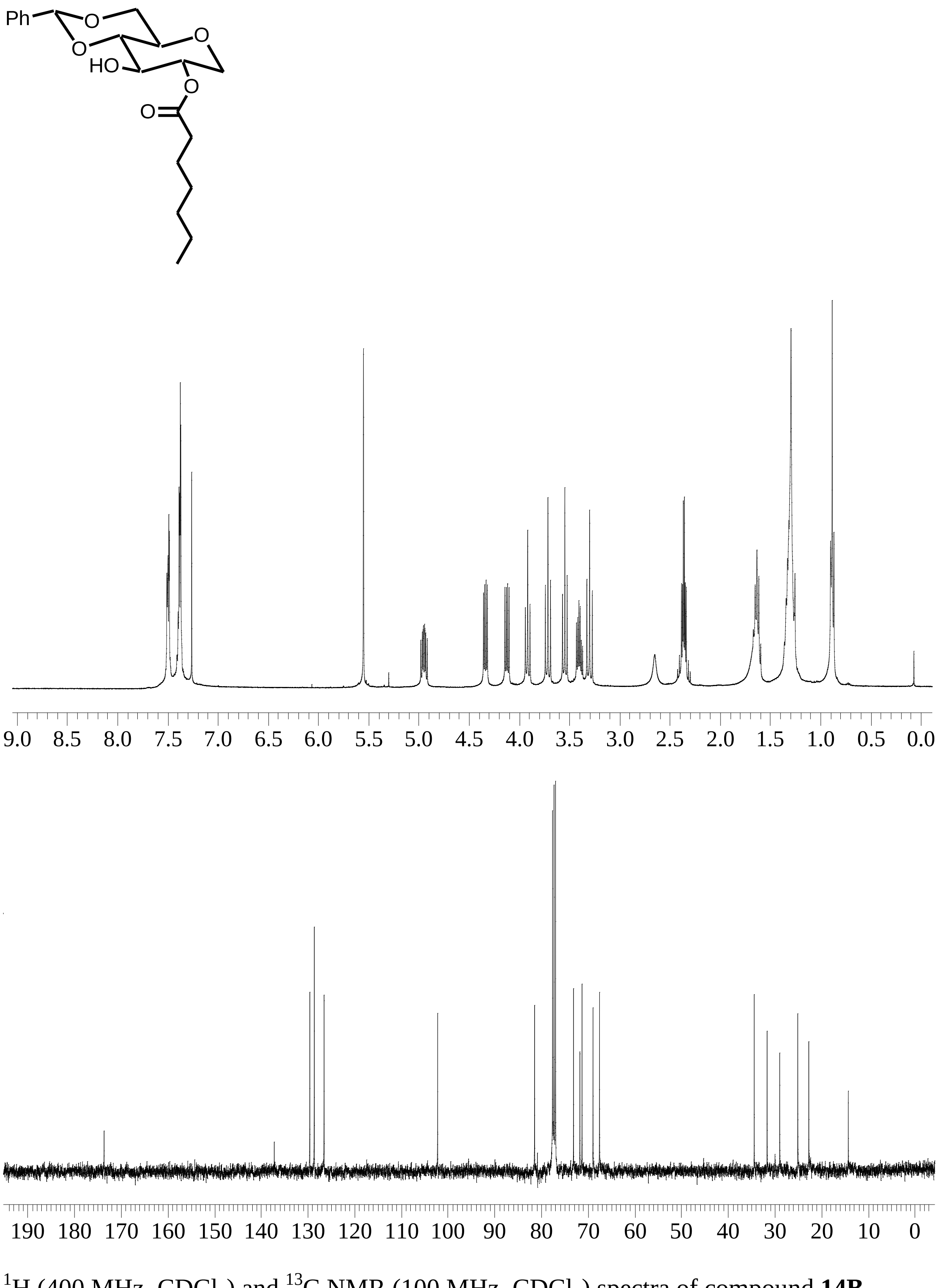

^{1}H (400 MHz, $CDCl_3$) and ^{13}C NMR (100 MHz, $CDCl_3$) spectra of compound **14B.**

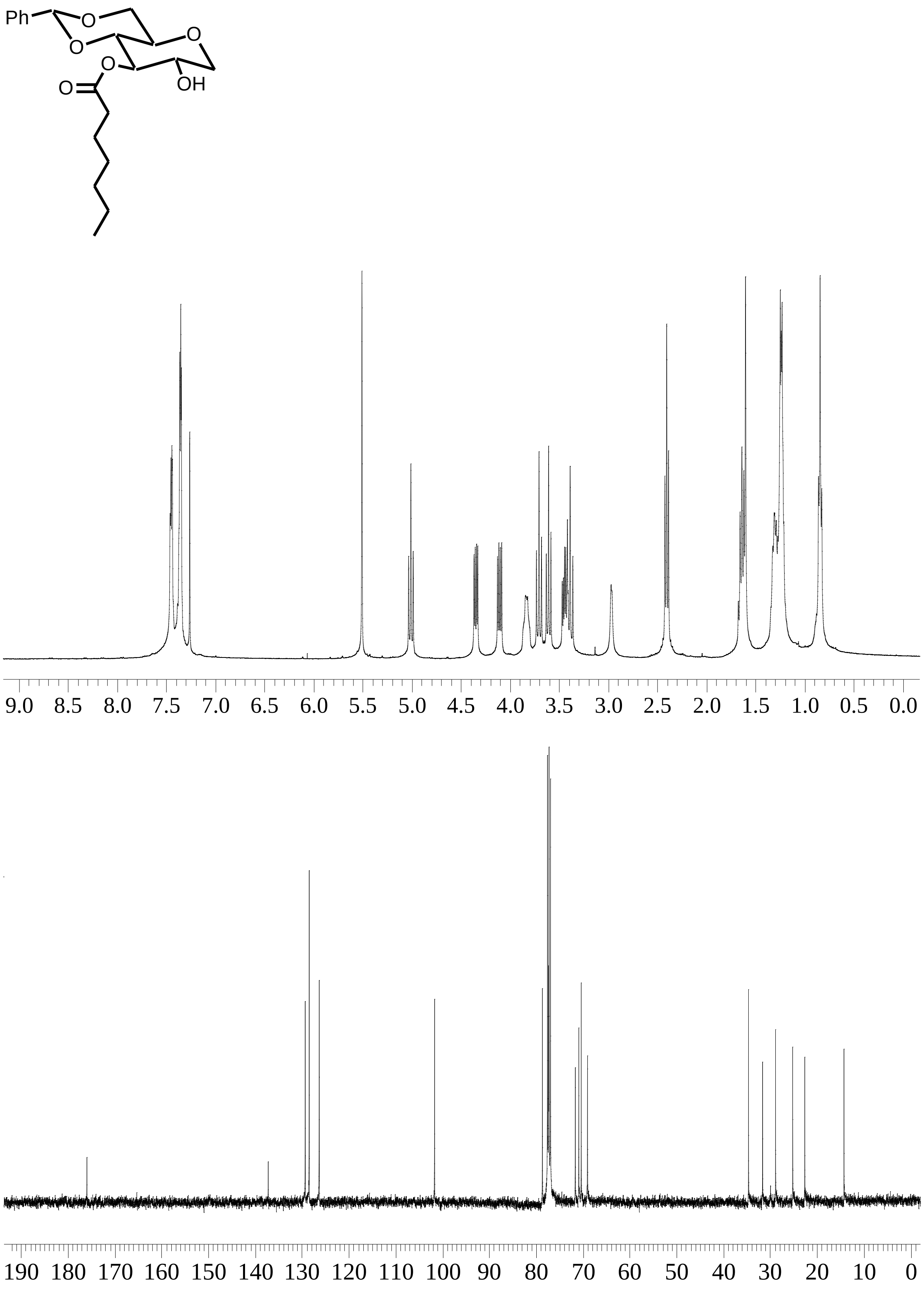

^{1}H (400 MHz, $CDCl_3$) and ^{13}C NMR (100 MHz, $CDCl_3$) spectra of compound **14C.**

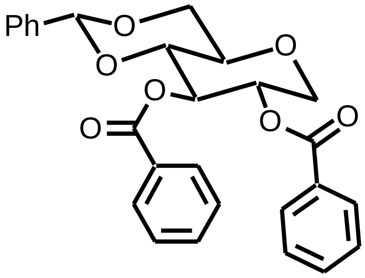

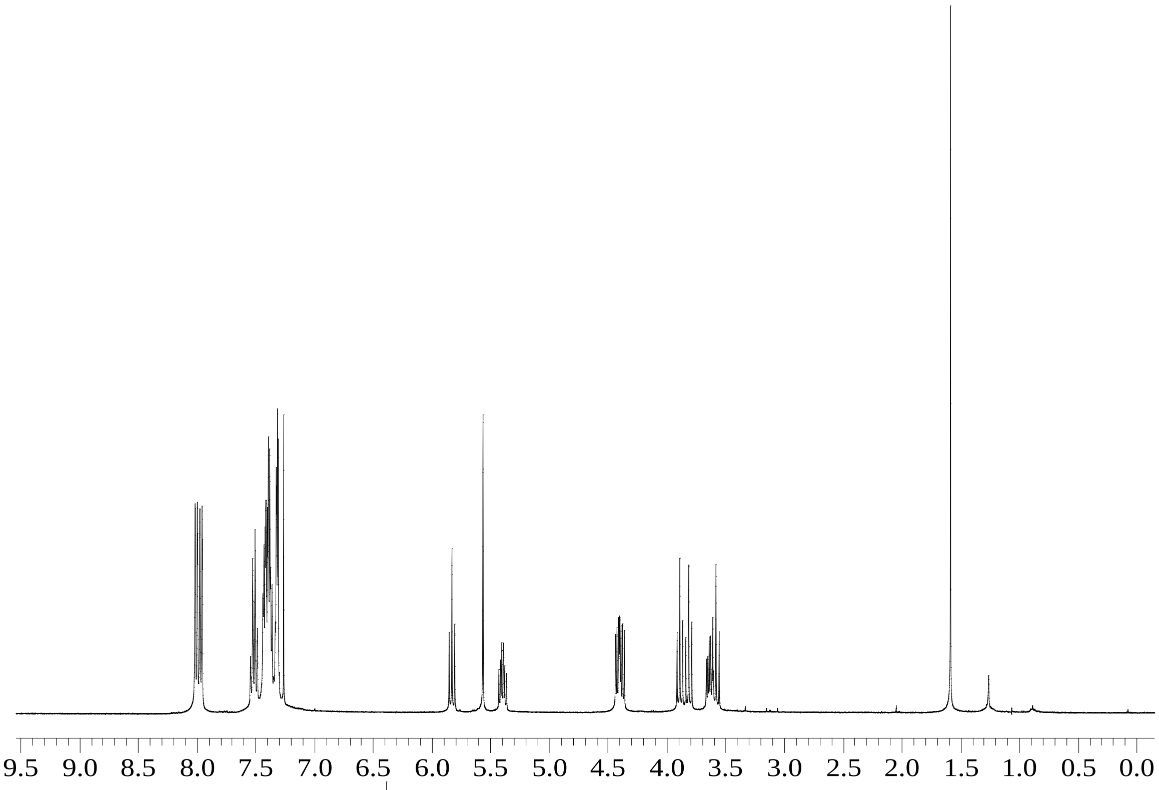

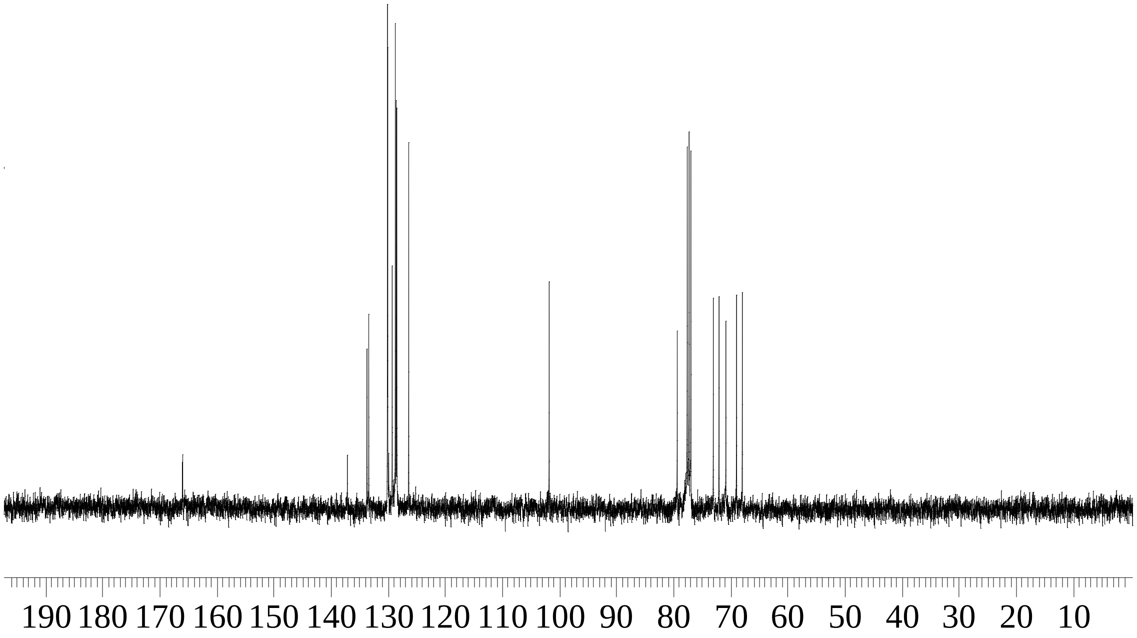

^{1}H (400 MHz, $CDCl_3$) and ^{13}C NMR (100 MHz, $CDCl_3$) spectra of compound **15A.**

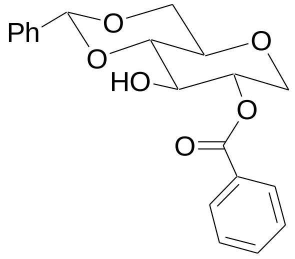

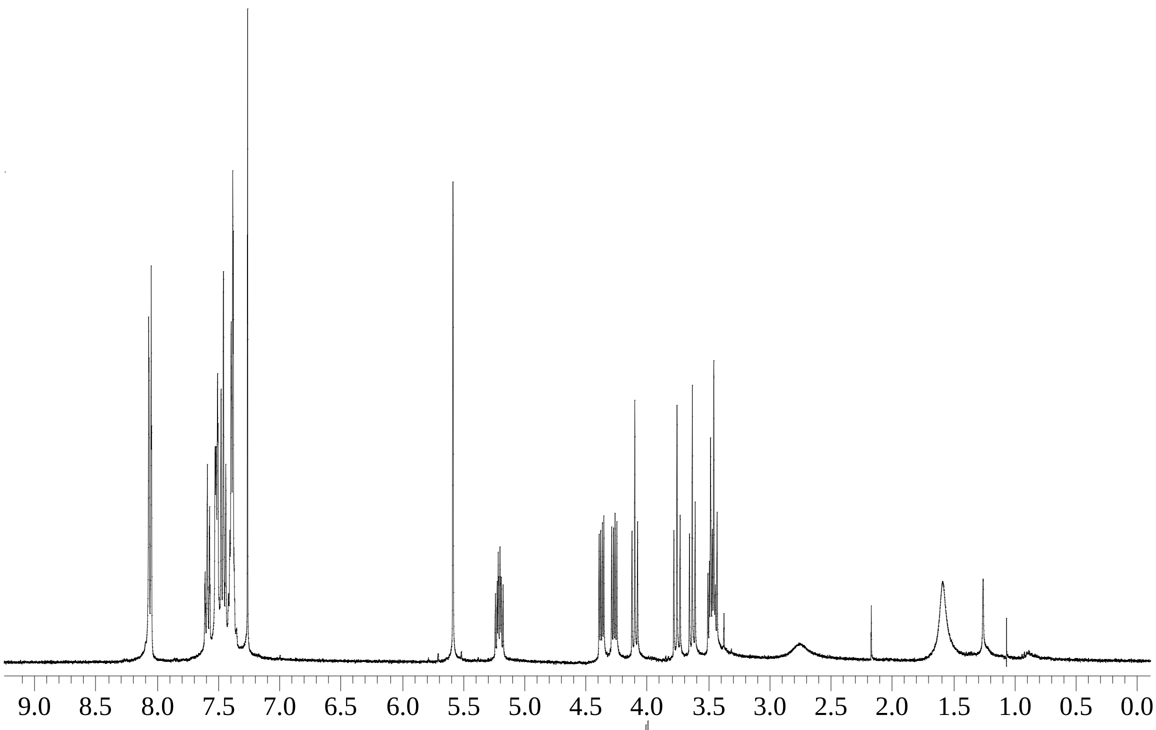

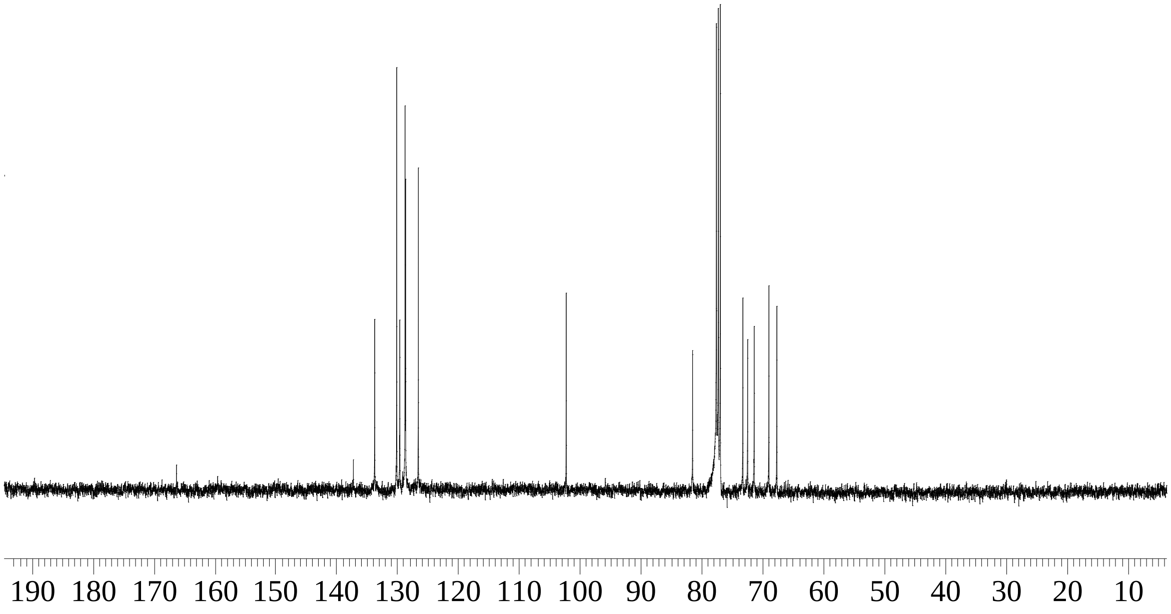

^{1}H (400 MHz, $CDCl_3$) and ^{13}C NMR (100 MHz, $CDCl_3$) spectra of compound **15B.**

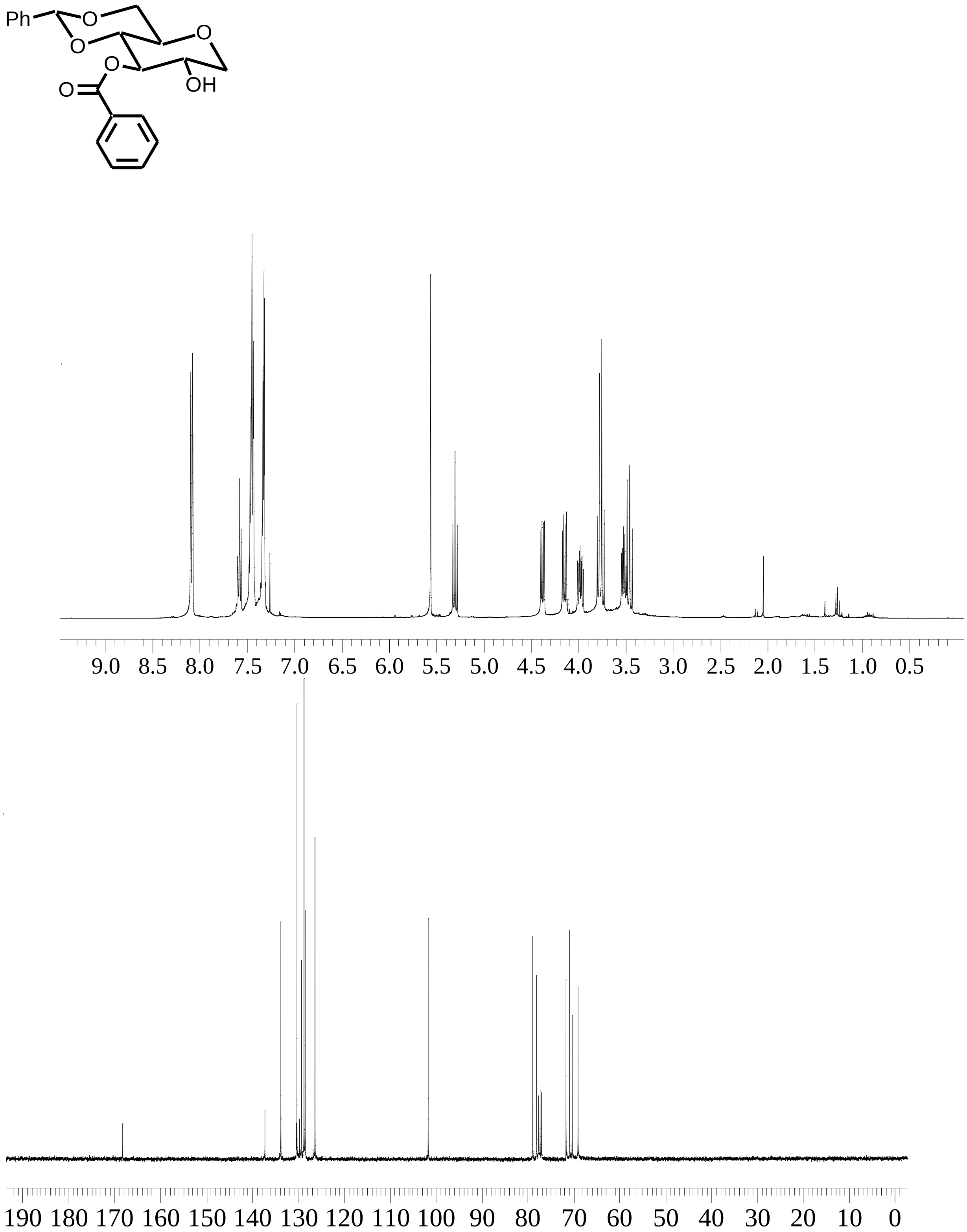

^{1}H (400 MHz, $CDCl_3$) and ^{13}C NMR (100 MHz, $CDCl_3$) spectra of compound **15C.**

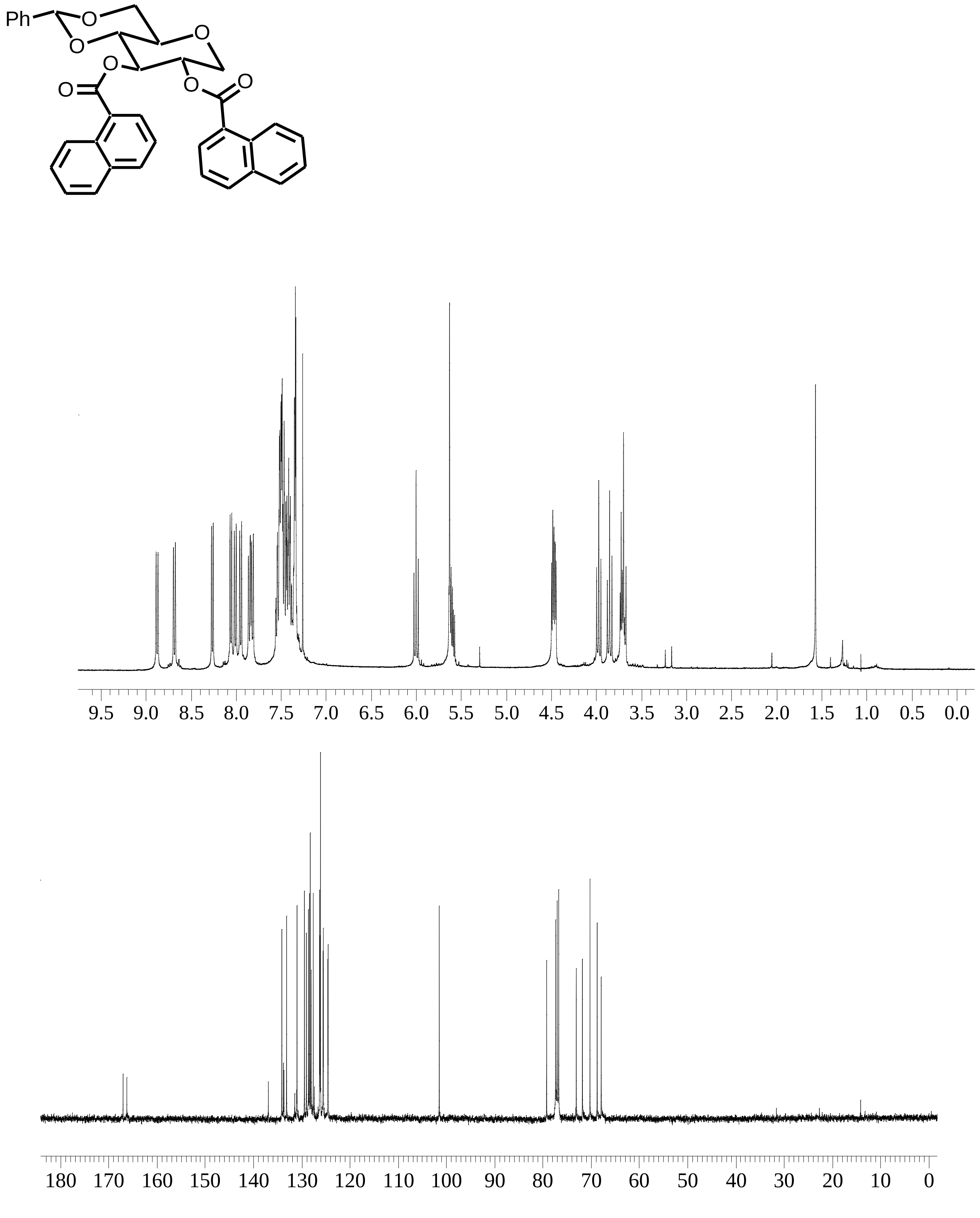

^{1}H (400 MHz, $CDCl_3$) and ^{13}C NMR (100 MHz, $CDCl_3$) spectra of compound **16A.**

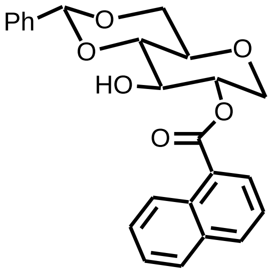

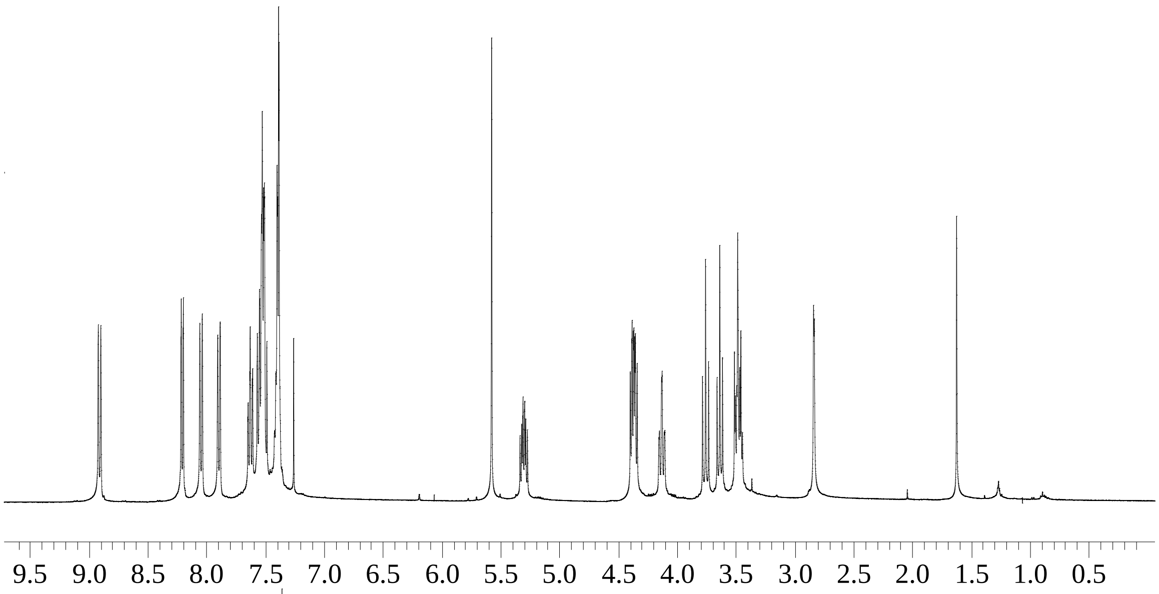

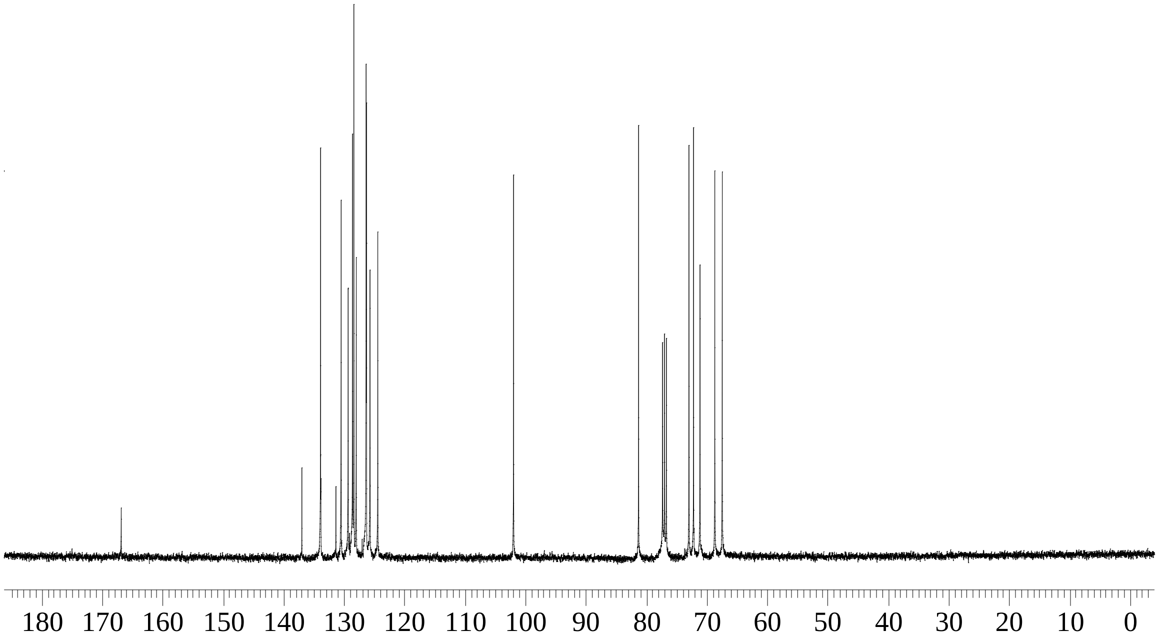

^{1}H (400 MHz, $CDCl_3$) and ^{13}C NMR (100 MHz, $CDCl_3$) spectra of compound **16B.**

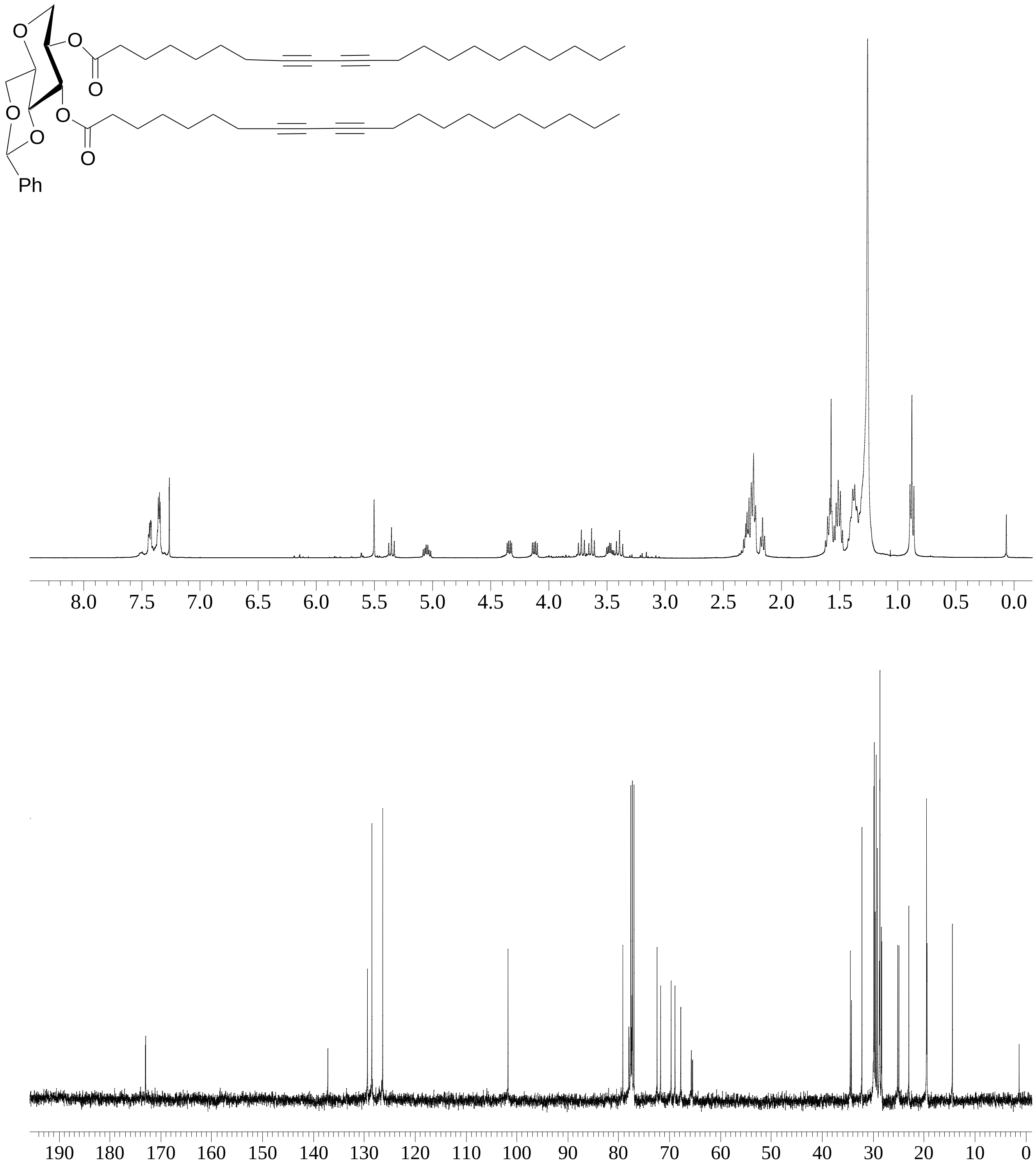

^{1}H (400 MHz, $CDCl_3$) and ^{13}C NMR (100 MHz, $CDCl_3$) spectra of compound **17A.**

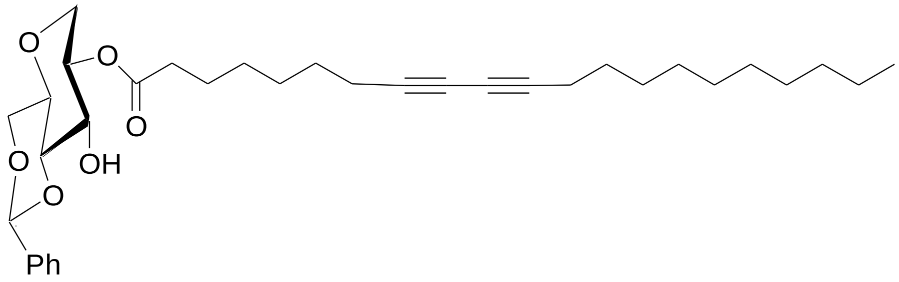

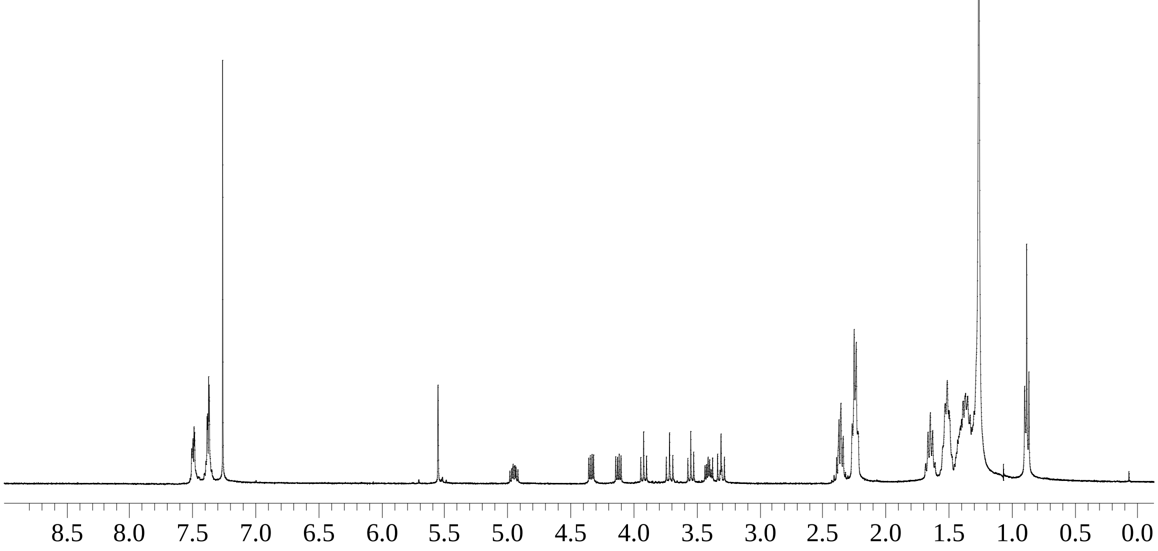

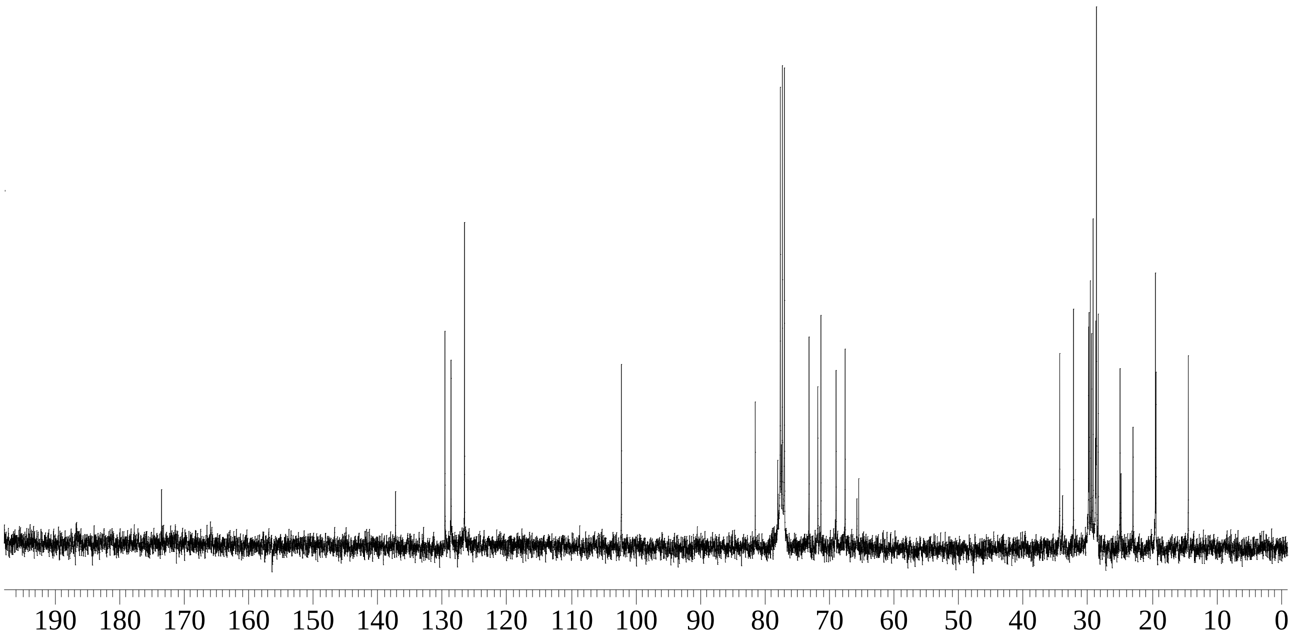

^{1}H (400 MHz, $CDCl_3$) and ^{13}C NMR (100 MHz, $CDCl_3$) spectra of compound **17B.**

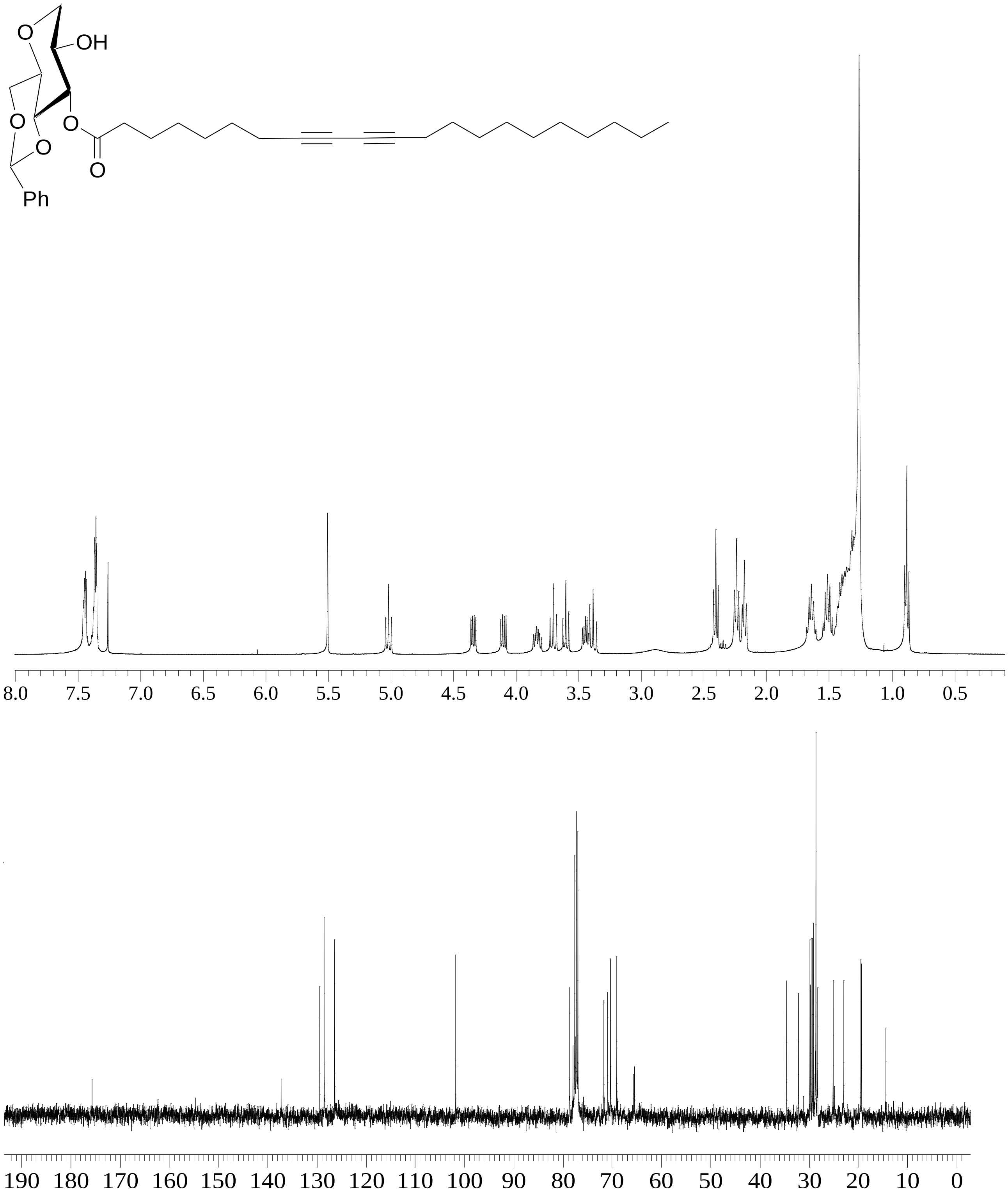

^{1}H (400 MHz, $CDCl_3$) and ^{13}C NMR (100 MHz, $CDCl_3$) spectra of compound **17C.**

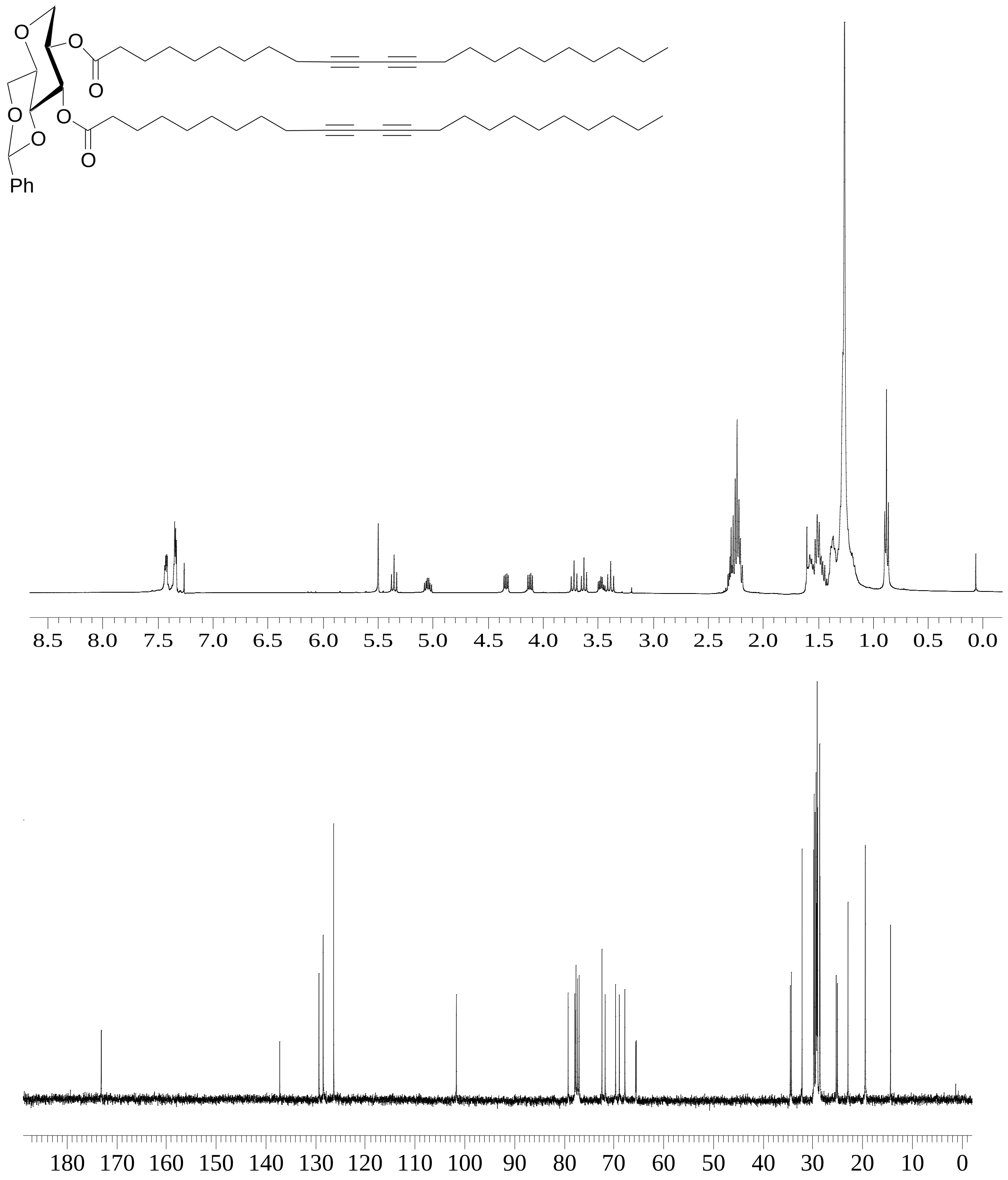

^{1}H (400 MHz, $CDCl_3$) and ^{13}C NMR (100 MHz, $CDCl_3$) spectra of compound **18A.**

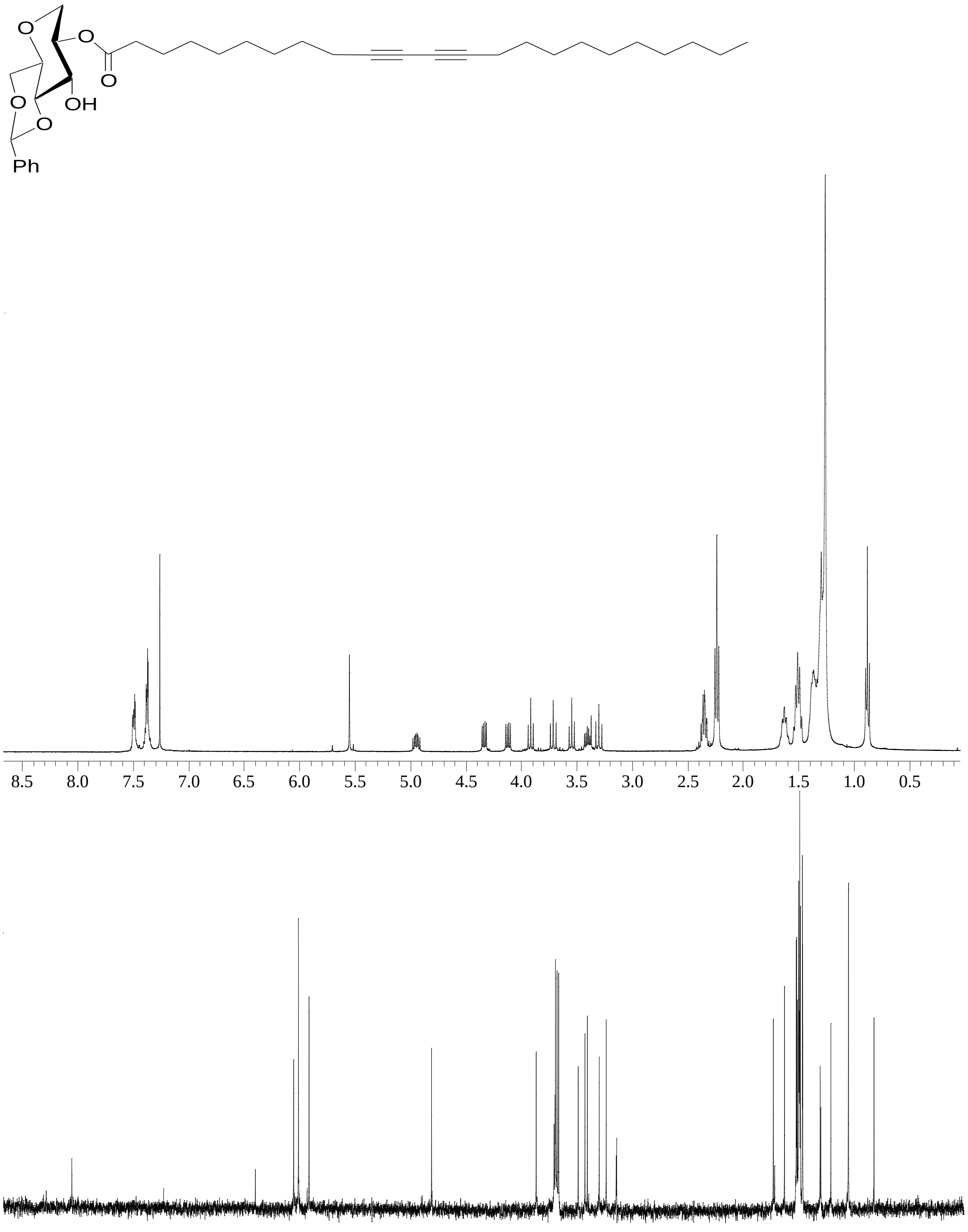

^{1}H (400 MHz, $CDCl_3$) and ^{13}C NMR (100 MHz, $CDCl_3$) spectra of compound **18B.**

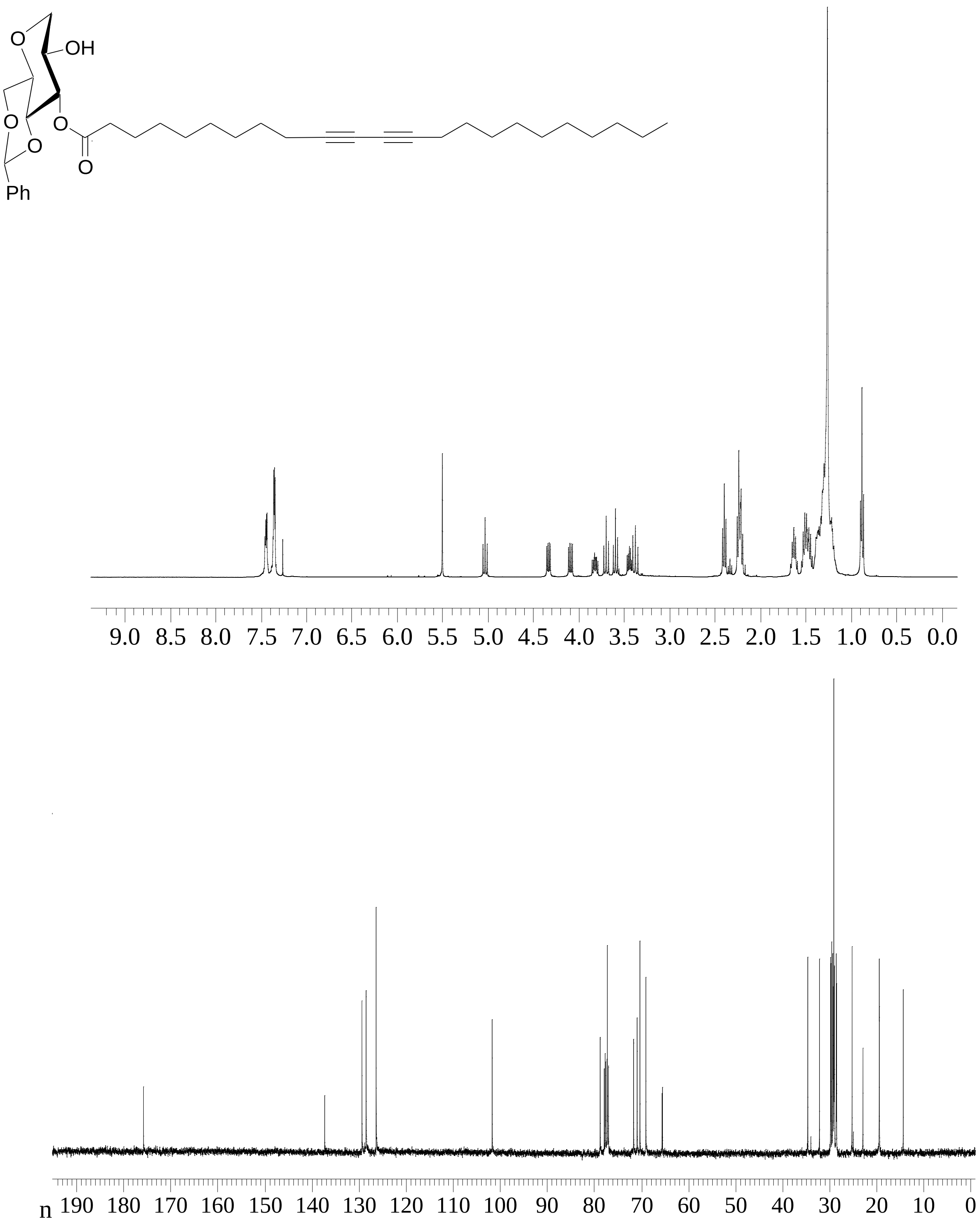

^{1}H (400 MHz, $CDCl_3$) and ^{13}C NMR (100 MHz, $CDCl_3$) spectra of compound **18C.**
